# Towards a Mathematical Theory of Cortical Micro-circuits

**DOI:** 10.1371/journal.pcbi.1000532

**Published:** 2009-10-09

**Authors:** Dileep George, Jeff Hawkins

**Affiliations:** Numenta Inc., Redwood City, California, United States of America; University College London, United Kingdom

## Abstract

The theoretical setting of hierarchical Bayesian inference is gaining acceptance as a framework for understanding cortical computation. In this paper, we describe how Bayesian belief propagation in a spatio-temporal hierarchical model, called Hierarchical Temporal Memory (HTM), can lead to a mathematical model for cortical circuits. An HTM node is abstracted using a coincidence detector and a mixture of Markov chains. Bayesian belief propagation equations for such an HTM node define a set of functional constraints for a neuronal implementation. Anatomical data provide a contrasting set of organizational constraints. The combination of these two constraints suggests a theoretically derived interpretation for many anatomical and physiological features and predicts several others. We describe the pattern recognition capabilities of HTM networks and demonstrate the application of the derived circuits for modeling the subjective contour effect. We also discuss how the theory and the circuit can be extended to explain cortical features that are not explained by the current model and describe testable predictions that can be derived from the model.

## Author Summary

Understanding the computational and information processing roles of cortical circuitry is one of the outstanding problems in neuroscience. In this paper, we work from a theory of neocortex that models it as a spatio-temporal hierarchical system to derive a biological cortical circuit. This is achieved by combining the computational constraints provided by the inference equations for this spatio-temporal hierarchy with anatomical data. The result is a mathematically consistent biological circuit that can be mapped to the cortical laminae and matches many prominent features of the mammalian neocortex. The mathematical model can serve as a starting point for the construction of machines that work like the brain. The resultant biological circuit can be used for modeling physiological phenomena and for deriving testable predictions about the brain.

## Introduction

Understanding the computational and information processing roles of cortical circuitry is one of the outstanding problems in neuroscience. The circuits of the neocortex are bewildering in their complexity and anatomical detail. Although enormous progress has been made in the collection and assimilation of data about the physiological properties and connectivity of cortical neurons, the data are not sufficient to derive a computational theory in a purely bottom-up fashion.

The theoretical setting of hierarchical Bayesian inference is gaining acceptance as the framework for understanding cortical computation [1]–[5]. Tai Sing Lee and David Mumford [1] suggested that algorithms for Bayesian belief propagation might model the interactive feed-forward and feedback cortical computations. Concurrently, Karl Friston [5] reviewed the structure of the anatomical organization of the neocortex and suggested its strong correspondence to hierarchical Bayesian generative models. Friston recently expanded on this to suggest an inversion method for hierarchical Bayesian dynamic models and to point out that the brain, in principle, has the infrastructure needed to invert hierarchical dynamic models [6]. However, there still remains a gap between our understanding of learning and inference in hierarchical Bayesian models and our understanding of how it is implemented in cortical circuits. In a recent review, Hegde and Felleman pointed out that the “Bayesian framework is not yet a neural model. [The Bayesian] framework currently helps explain the computations that underlie various brain functions, but not how the brain implements these computations” [2]. This paper is an attempt to fill this gap by deriving a computational model for cortical circuits based on the mathematics of Bayesian belief propagation in the context of a particular Bayesian framework called Hierarchical Temporal Memory (HTM).

Belief propagation techniques can be applied to many different types of networks. The networks can vary significantly in their topology, in how they learn (supervised, unsupervised, or non-learning), and in how they incorporate or do not incorporate time. Therefore, to map the mathematics of Bayesian belief propagation onto cortical architecture and microcircuits we must start with a particular Bayesian framework that specifies these variables. The starting point for the work presented in this paper is a model called the Memory-Prediction Framework, first described by one of this paper's authors, Hawkins, in a book titled “On Intelligence” [7]. The Memory-Prediction Framework proposed that the neocortex uses memory of sequences in a hierarchy to model and infer causes in the world. The Memory-Prediction Framework proposed several novel learning mechanisms and included a detailed mapping onto large scale cortical-thalamic architecture as well as onto the microcircuits of cortical columns. However, the Memory-Prediction Framework was not described in Bayesian terms and was presented without the rigor of a mathematical formulation.

This paper's other author, George, recognized that the Memory-Prediction framework could be formulated in Bayesian terms and given a proper mathematical foundation [8],[9]. We call this formulation Hierarchical Temporal Memory (HTM) and it is currently being applied to problems of machine learning and inference. The final step in this theory is to map the mathematics of HTM directly to cortical-thalamic anatomy and the microcircuits of cortical columns. That is the goal of this paper. We will work back from the formal expression of HTM and derive cortical microcircuits by matching the computational specifications of the theory with known biological data. The resultant biological circuit supports all the Bayesian computations required for temporal, feed-forward, and feedback inference. The elements of the circuits are also consistent with each other in that they operate under the same set of assumptions and work together in a hierarchy.

Several researchers have proposed detailed models for cortical circuits [10]–[12]. Some of these models exhibit interesting pattern recognition properties and some have been used in the explanation of physiological phenomena. However, these models do not incorporate the concepts of Bayesian inference in a hierarchical temporal model. Other researchers [4],[13] have proposed detailed mechanisms by which Bayesian belief propagation techniques can be implemented in neurons. Their work suggests that, at a neuron level, machinery exists for implementing the types of computations required for belief propagation. However, they did not attempt to map these implementations to detailed cortical anatomy. To our knowledge, the work in this paper is the first attempt to map the theory of Bayesian belief propagation and hierarchical and temporal inference onto cortical circuitry. (Partial details of this work have been published earlier [9],[14].)

Deciphering the functional connectivity of the cortical circuits is a formidable task and is associated with the perils involved in the reverse engineering of a complex system. The circuits derived in this chapter can provide a hypothesis-driven framework for examining the neural connectivity. As with any theory, it is expected that the particular instantiation described here will need to be revised as more data is obtained and more aspects of cortical computations, like attention, timing, and motor action, are incorporated. The circuit derived here could act as a basis for such explorations. In addition to providing a template for understanding cortical circuits [15], the theory presented here can be useful in the modeling of physiological phenomena. As an example, we simulate the subjective contour effect using feedback from a high-level belief using the derived circuits. Having a complete biological mapping of a computational theory can also help in the design of hypothesis-driven biological experiments.

The rest of this paper is organized in such a manner that the computational parts are clearly separated from the biological aspects. The Model section deals exclusively with the computational aspects of HTMs. In this section, we briefly describe the HTM theory and take a detailed look at the inference mechanism in HTM nodes. The Bayesian belief propagation equations for the computations in an HTM node are described. We then describe an abstract circuit implementation of these equations using neuron-like elements. The Results section of the paper, which deals primarily with the biological implementation, maps this abstract neural implementation to the laminar biological cortical circuitry by matching the computational specifications with anatomical data. This section also provides example applications of this circuit in the modeling of physiological phenomena. In the Discussion section we discuss variations, omissions, and extensions of the proposed circuits.

## Model

### Hierarchical Temporal Memory

Hierarchical Temporal Memory is a theory of the neocortex that postulates that the neocortex builds a model of the world using a spatio-temporal hierarchy. According to this theory, the operation of the neocortex can be approximated by replicating a basic computational unit – called a node – in a tree structured hierarchy. Each node in the hierarchy uses the same learning and inference algorithm, which entails storing spatial patterns and then sequences of those spatial patterns. The feed-forward output of a node is represented in terms of the sequences that it has stored. The spatial patterns stored in a higher-level node record co-occurrences of sequences from its child nodes. The HTM hierarchy is organized in such a way that higher levels of the hierarchy represent larger amounts of space and longer durations of time. The states at the higher levels of the hierarchy vary at a slower rate compared to the lower levels. It is speculated that this kind of organization leads to efficient learning and generalization because it mirrors the spatio-temporal organization of causes in the world.

In our research, HTMs have been used successfully in invariant pattern recognition on gray-scale images, in the identification of speakers in the auditory domain and in learning a model for motion capture data in an unsupervised manner. Other researchers have reported success in using HTMs in content-based image retrieval [16], object categorization [17], and power system security analysis [18]. Another set of researchers has explored hardware implementations and parallel architectures for HTM algorithms [19].

**Figure 1:**
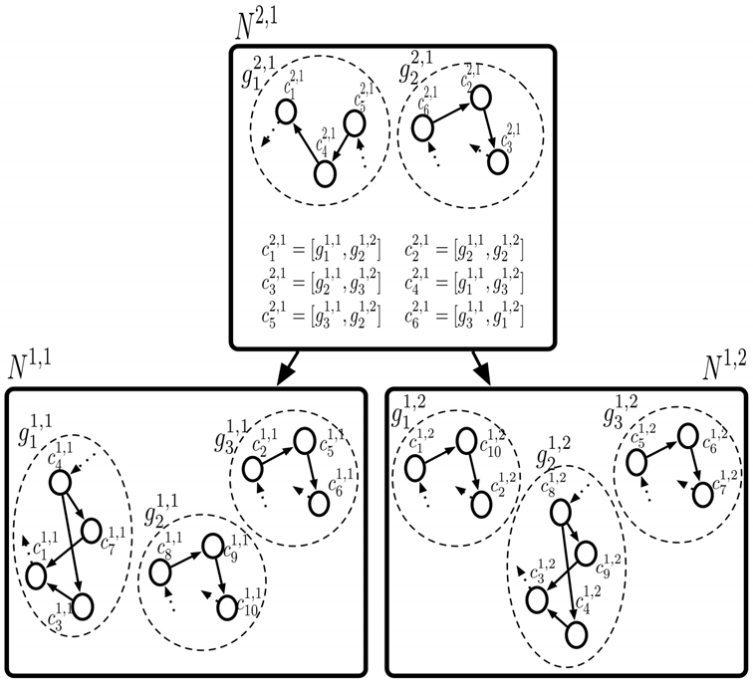
Generative model for HTM. Hierarchical Temporal Memory (HTM) is a model of neocortical function. HTMs can be specified using a generative model. Shown is a simple two-level three-node HTM-type generative model. Each node in the hierarchy contains a set of coincidence patterns (labeled with 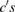) and a set of Markov chains (labeled with 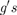) defined over the set of coincidence patterns. A coincidence pattern in a node represents a co-activation of particular Markov chains of its child nodes. HTM generative model is a spatio-temporal hierarchy in which higher levels remain stable for longer durations of time and can generate faster changing activations in lower levels.

HTMs can be specified mathematically using a generative model. A simplified two-level generative model is shown in Figure 1. Each node in the hierarchy contains a set of *coincidence patterns* 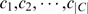 and a set of Markov chains 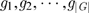 where each Markov chain is defined over a subset of the set coincidence patterns in that node. A coincidence pattern in a node represents a co-activation of the Markov chains of its child nodes. A coincidence pattern that is generated by sampling a Markov chain in a higher level node concurrently activates its constituent Markov chains in the lower level nodes. For a particular coincidence pattern and Markov chain that is ‘active’ at a higher-level node, sequences of coincidence patterns are generated concurrently by sampling from the activated Markov chains of the child nodes.

The process of learning an HTM model for spatio-temporal data is the process of learning the coincidence patterns and Markov-chains in each node at every level of the hierarchy. Although algorithms of varying levels of sophistication can be used to learn the states of an HTM node, the basic process can be understood using two operations, (1) memorization of coincidence patterns, and (2) learning a mixture of Markov chains over the space of coincidence patterns. In the case of a simplified generative model, an HTM node remembers all the coincidence patterns that are generated by the generative model. In real world cases, where it is not possible to store all coincidences encountered during learning, we have found that storing a fixed number of a random selection of the coincidence patterns is sufficient as long as we allow multiple coincidence patterns to be active at the same time. Motivation for this method came from the field of compressed sensing [20]. The HMAX model of visual cortex [21] and some versions of convolutional neural networks [22] also use this strategy. We have found that reasonable results can be achieved with a wide range of the number of coincidences stored. We have not yet developed a good heuristic for determining an optimal value of this parameter. For simplicity, we will only illustrate the case where a single coincidence pattern is active in a node at a time, but in our real implementations we use sparse distributed activations of the coincidence patterns. Each Markov chain in a node represents a set of coincidence patterns that are likely to occur sequentially in time. This temporal proximity constraint is analogous to the temporal slowness principle used in the learning of of invariant features [23]–[26]. The learning of the mixture of Markov chains is simplified considerably because of the slowness constraint. We have found that a simple way to learn the mixture of Markov chains for real world cases is to learn a large transition matrix that is then partitioned using a graph partitioning algorithm [27]. Details of one method of learning higher order Markov chains is available in [28].

**Figure 2:**
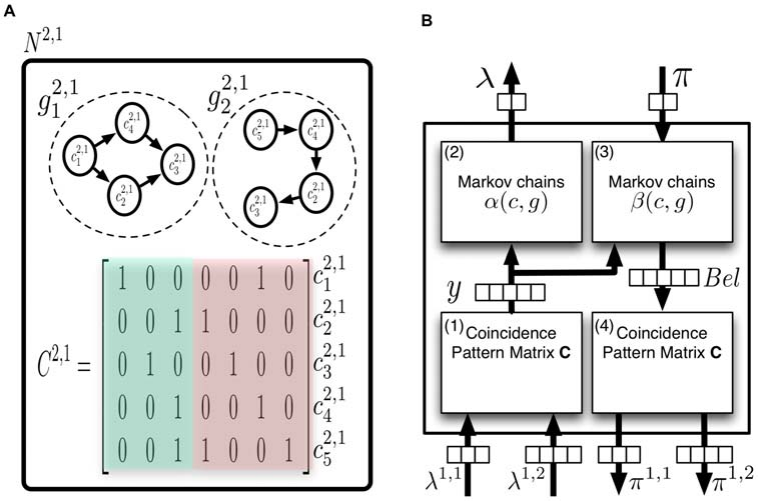
Structure and flow of a reference HTM node. (A) Structure of the reference node, with five coincidence patterns and two Markov chains. This is an HTM node that has finished its learning process. It is assumed that this is the first node at level 2 of a network and is therefore labeled as 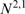. Each coincidence pattern represents a co-occurrence of the Markov chains of the children. This node has 2 children. Child 1 has 3 Markov chains and child 2 has 4 Markov chains – hence there are seven elements in each coincidence pattern. The portions of the coincidence pattern coming from the first and second child are shown in different shades of gray. (B) Information flow in the reference node for the computation of the belief propagation equations shown in Table 1. The rectangles inside the node are processing units for the equations in the rows corresponding to the number displayed in each rectangle. We will use ‘feed-forward’ or ‘bottom-up’ to qualify messages received from children and messages sent up to the parent of this node. We will use ‘feedback’ or ‘top-down’ to qualify messages received from the parent and messages sent to the child nodes of this node. The node shown in the figure has two bottom-up input messages coming from the two children and has two top-down outputs which are the messages sent to these children. The arrows show vectors of inputs, outputs, and intermediate computational results. The number of components of each vector is represented using an array of boxes placed on these arrows.

For the rest of this paper, we will focus on the inference mechanism in HTM nodes that have finished their learning process. A node that has finished its learning process has a set of coincidence patterns and a set of Markov chains in it. Figure 2(A) shows a node that has 5 coincidence patterns and 2 Markov chains.

The inference mechanism in an HTM network is based on the propagation of new evidence from anywhere in the network to all other parts of the network. The presentation of a new image to the first level of an HTM vision network is an example of new evidence. Propagation of this evidence to other parts of the network results in each node in the network adjusting its belief states given this evidence. For example, a new image can lead to a different belief in the top level of the network regarding the identity of the object in that image. In general, HTM networks infer on time-varying inputs. Inference on a static input is a special case of this computation. Information can also be propagated down in the hierarchy for attention, segmentation, and filling in missing inputs.

HTM networks use Bayesian belief propagation for inference. Bayesian belief propagation originally was derived for inference in Bayesian networks [29]. Since an HTM node abstracts space as well as time, new equations must be derived for belief propagation in HTM nodes. These equations are described in the next section.

### Belief propagation in HTM nodes

In general, the messages that come into an HTM node from its children represent the degree of certainty over the child Markov chains. The node converts these messages to its own degree of certainty over its coincidence patterns. Based on the history of messages received, it also computes a degree of certainty in each of its Markov chains. This is then passed up to the next higher-level node. What the node receives from its parent is the parent's degree of certainty over this HTM node's Markov chains. The Markov chains are then ‘unwound’ in a step-by-step manner to find the top-down probability distribution over coincidence patterns. From this, the node's degrees of certainty over its child nodes' Markov chains are calculated. These feedback messages are then sent to the child nodes.

Table 1 summarizes the computation of belief propagation messages in an HTM node. We will now describe the notation and meaning of these equations using the reference HTM node shown in Figure 2. Detailed derivations of these equations are given in supporting information Text S1. A summary of the notation in these equations is given in Table 2. Each equation is considered in detail in the sections that follow.

**Table 1:** Belief propagation equations for an HTM node.

1) Calculate likelihood over coincidence patterns.	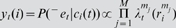 (2) where coincidence pattern 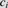 is the co-occurrence of 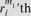 Markov chain from child 1, 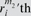 Markov chain from child 2, 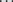 , and 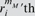 Markov chain from child 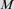 .
2) Calculate the feed-forward likelihood of Markov chains using dynamic programming	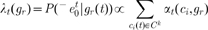 (3) 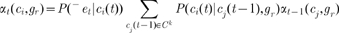 (4) 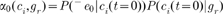 (5)
3) Calculate the belief distribution over coincidence patterns	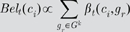 (6) 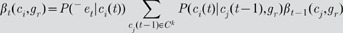 (7) 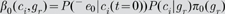 (8)
4) Calculate the messages to be sent to child nodes.	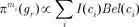 (9) where 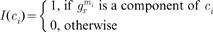 (10)

**Table 2:** Summary of notation used for belief propagation in HTM nodes.

Symbol	Meaning
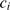	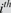 coincidence in the node
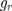	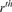 Markov chain in the node.
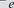	Bottom-up evidence. 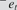 indicates the evidence at particular instant 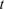 and 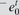 indicates the sequence of bottom-up evidence from time 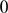 to time 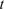 .
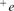	Top-down evidence. Time indexing is similar to that of 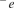
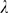	Feed-forward output message of the node. This is a vector of length equal to the number of Markov chains in the node.
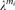	Feed-forward input message to the node from the child node 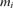 . This is a vector of length equal to the number of Markov chains in the child node.
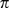	Feedback input message to the node. This is a vector of length equal to the number of Markov chains in the node.
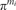	Feedback output message of the node to child node 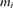 . This is a vector of length equal to the number of Markov chains in the child node.
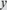	The bottom-up likelihood over coincidence patterns in a node. This is one of the inputs for the feed-forward sequence likelihood calculation.
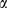	Bottom-up state variable for the Markov chains in a node. This a vector of length equal to the total number of states of all Markov chains in the node.
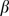	State that combines bottom-up and top-down evidence for the Markov chains in a node. This state variable has the same dimension as that of 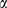 .
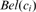	Belief in the 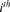 coincidence pattern in a node.

In these equations, the coincidence patterns are referred to using 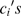 and the Markov chains are referred to using 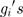. The HTM node shown in Figure 2(A) contains 5 coincidence patterns and 2 Markov chains. The transition probability matrix of the Markov chain 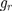 is denoted by 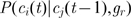. This term appears in Equations 4 and 7. Each coincidence pattern in the node represents a co-occurrence of the temporal groups from its children. Coincidence pattern specifications are used in the computations described in equations 2 and 9.

Each node receives feed-forward input messages from its children and sends feed-forward messages to its parent. The feed-forward input messages are denoted by 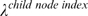. The feed-forward output message of the node is denoted by 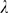. Similarly, the node receives feedback messages from its parent and sends feedback messages to its child nodes. The feedback input message to the node is denoted by 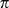. The feedback output messages that the node sends to its child nodes are denoted by 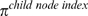. The equations shown in Table 1 describe how the output messages are derived from the input messages. From the viewpoint of the node, the feed-forward messages carry information about the *evidence* from below. Evidence from below at any time 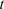 is denoted by 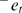. Similarly evidence from the parent is denoted by 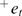.

Equation 2 describes how the node calculates its likelihood of coincidence patterns, using the messages it gets from the children. The bottom-up likelihood of coincidence pattern 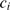 at time 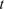 is represented by 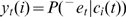. The likelihood of each coincidence pattern is calculated as the product of the message components corresponding to that coincidence pattern.

In Equation 3, the bottom-up likelihood of Markov chain 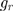 at time 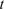 is denoted by 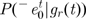, where the term 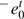 represents the sequence of bottom-up evidences from time 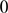 to time 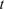. This reflects that the likelihood of the Markov chains depends on the sequence of inputs received by the node. The variables 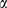 and 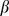 defined in Equations 4 and 7 are state variables that are updated in a recursive manner at every time instant. These are dynamic programming [30],[31] variables, each defined over all pairwise combinations of coincidence patterns and Markov chains. For example, 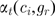 is value of the feed-forward dynamic programming variable at time 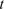 corresponding to coincidence 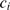 and Markov chain 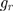. In Equations 4 and 7, the states are updated every time step by passing the state from the previous time step through the Markov transition matrices and by combining them with bottom-up/top-down evidence.

An illustrative example showing how the belief propagation equations map onto a toy visual pattern recognition problem is given in supporting information Text S2. Readers who are not familiar with belief propagation can use this example to develop intuition for the nature of the messages. We examine the equations in Table 1 in more detail in the next section as we consider how to implement them using neuron-like elements.

### Neuronal implementation of HTM belief propagation

This section describes an implementation of the HTM belief propagation equations using neuron-like elements. The implementation will be described with respect to the reference HTM node in Figure 2. The neuronal implementation of the equations in Table 1 is described in the following subsections. The subsections follow the order of table row numbers.

The purpose of this section is to show how the equations of HTM belief propagation can map onto a hypothetical neuronal system. In the Results section, we map this hypothetical model onto actual cortical anatomy.

#### Calculating the likelihood of coincidence patterns

**Figure 3:**
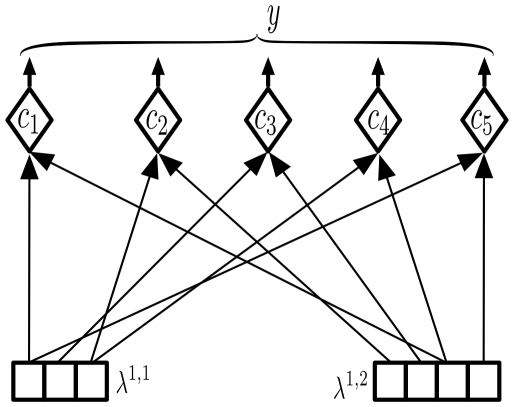
Coincidence likelihood circuit. Circuit for calculating the bottom-up probability over coincidence patterns. Coincidence pattern neurons are represented by diamond shapes. The inputs to the circuit are the messages from the children, which are denoted by 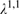 and 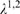. The output of the circuit is 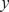, as calculated by Equation 2 in Table 1. The input connections to each neuron represent its coincidence pattern. For example, 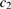 is the co-occurrence of Markov chain 3 from the left child and Markov chain 1 from the right child. The probabilities are calculated by multiplying the inputs to each neuron.

The bottom-up input to the HTM node is the feed-forward output messages from its children. These output messages carry information about the degree of certainty of the Markov chains in the child nodes. Each message is a vector of length equal to the number of Markov chains in the corresponding child. The likelihood of coincidences is derived from these input messages according to Equation 2. This operation is performed by the rectangle marked 1 in Figure 2(B). Figure 3 shows an abstract neuronal implementation of this calculation for the reference HTM node.

In Figure 3, each neuron corresponds to a stored coincidence pattern. The pattern corresponding to the co-occurrence is stored in the connections this neuron makes to the messages from the child input nodes. For example, the neuron corresponding to coincidence pattern 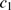 has connections to the first position of the message from the first child and the third position of the message from the second child. These connections correspond to first row of the coincidence-pattern matrix 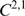 in Figure 2(A). Each neuron calculates its output by multiplying its inputs. For example, the output of neuron 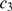 is proportional to the product of 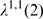 and 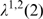. The output, denoted by 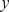 in Figure 2(B), is a vector of 5 components, one component corresponding to each coincidence pattern. This vector represents the likelihood of coincidence patterns, based on the messages received from the child nodes.

#### Calculating the feed-forward likelihood of Markov chains

The next step in the computation of feed-forward messages, corresponding to the rectangle marked 2 in Figure 2(B), is the calculation of the degree of certainty of the HTM node in each of its Markov chains. The quantity that needs be to calculated is 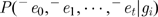 for each Markov chain 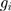 where 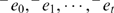 represent the bottom-up evidence distributions received from time 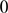 to time 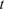. The likelihood of Markov chains depends on the sequence of messages that the node has received from its children. A brute-force computation of this quantity is not feasible because this requires the enumeration of the likelihoods of an exponentially growing number of sample paths. To calculate 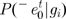 efficiently, all the past evidence needs to be collapsed into a state variable that can be updated recursively every time instant. This is done using a technique called dynamic programming [30],[31] as represented in Equation 4. The derivation of this equation is described in supporting information Text S1.

**Figure 4:**
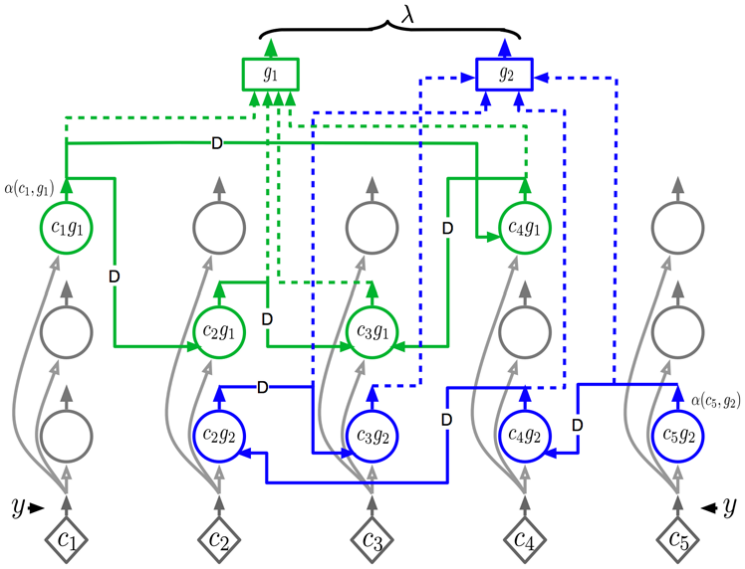
Markov chain likelihood circuit. The circuit for calculating the likelihoods of Markov chains based on a sequence of inputs. In this figure there are five possible bottom-up input patterns (c1–c5) and two Markov chains (g1, g2). The circle neurons represent a specific bottom-up coincidence within a learned Markov chain (two Markov chains are shown, one in blue and one in green). Each rectangular neuron represents the likelihood of an entire Markov chain to be passed to a parent node. This circuit implements the dynamic programming Equation 4 in Table 1.

Equation 4 can have a very efficient neuronal implementation as shown in Figure 4. The ‘circle’ neurons in this circuit implement the sequence memory of the Markov chains in the HTM node. The connections between the circle neurons implement the transition probabilities of the Markov chain. As the ‘axons’ between these neurons encode a one time-unit delay, the output of a circle neuron is available at the input of the circle neuron that it connects to after one time step. (This is a very limited method of representing time. We will discuss more sophisticated representations of time in a later section.)

All the circle neurons co-located in a column have the same bottom-up input. They are all driven by the same coincidence-pattern likelihood neuron – represented by diamonds – from below. Each column, considering only bottom-up input, can be thought of as representing a particular coincidence pattern. In addition to the bottom-up input, these circle neurons also have ‘lateral’ inputs that come from other circle neurons in the same Markov chain. The lateral connections specify the meaning of a neuron in a sequence. A circle neuron that is labeled as 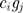 represents the coincidence pattern 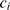 in the context of Markov chain 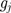. The same coincidence pattern can belong to different Markov chains and can hence be active under different temporal contexts. For example, the circle neuron 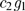 will be activated only in the context of Markov chain 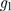, whereas the circle neuron 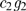 will be activated only in the context of Markov chain 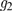.

Each circle neuron in this circuit does the same computation. Each neuron calculates its output by multiplying the bottom-up input with the weighted sum of its lateral inputs. The output of a circle neuron is denoted using 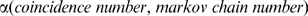. With this, the output of any circle neuron 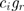 is calculated as
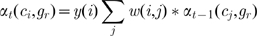
(1)That is, the output of the a circle neuron at any time is the weighted sum of the outputs of the neurons in the same Markov chain at the previous time step multiplied by the current bottom-up activation. (Again, the above equation assumes a simple step-wise notion of time which is insufficient for encoding duration and for non-discrete time problems. We believe that in real brains, time duration is captured using a separate mechanism. This will be discussed in the Results section.) The above equation corresponds to Equation 4 if we replace 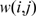 by 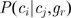. Therefore, the circle-neuron circuits shown in Figure 4 implement Equation 4 and the weights on the lateral time-delayed connections correspond to the transition matrix entries in each Markov chain.

Now consider the third kind of neurons – the ‘rectangle’ neurons – in Figure 4. The rectangle neuron marked 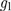 receives its inputs from the outputs of all the circle neurons in the Markov chain 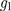. The rectangle neurons pool the outputs of all the coincidence-pattern neurons in the context of a Markov chain. At any time point, the output of a rectangle neuron is calculated as the sum (or maximum) of the inputs to that neuron.

Note that the operation of the rectangle neurons corresponds to pooling over the activations of all the circle neurons of the same Markov chain. It is easy to verify that this is the operation involved in the calculation of the message this node sends to its parent according to Equation 3. The concatenated outputs of the rectangle neurons is the message 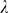 that this node sends to its parent. As noted in Figure 2(B), this message is a vector of two components, corresponding to the two Markov chains in the reference node in Figure 2(A). This completes the description of the abstract neuronal implementation of equations in the second row of Table 1 and of the operations performed by the rectangle marked (2) in Figure 2(B).

#### Calculating the belief distribution over coincidence patterns

An HTM node calculates its degree of belief in a coincidence pattern by combining bottom-up, top-down, and temporal evidences according to the equations on the third row of Table 1. This corresponds to the operations of the rectangle marked (3) in Figure 2(B). The top-down input to the node is a vector of length equal to the number of Markov chains of the node. The output of this computation is the belief-vector over the coincidence patterns, in this case, a vector of length 5.

**Figure 5:**
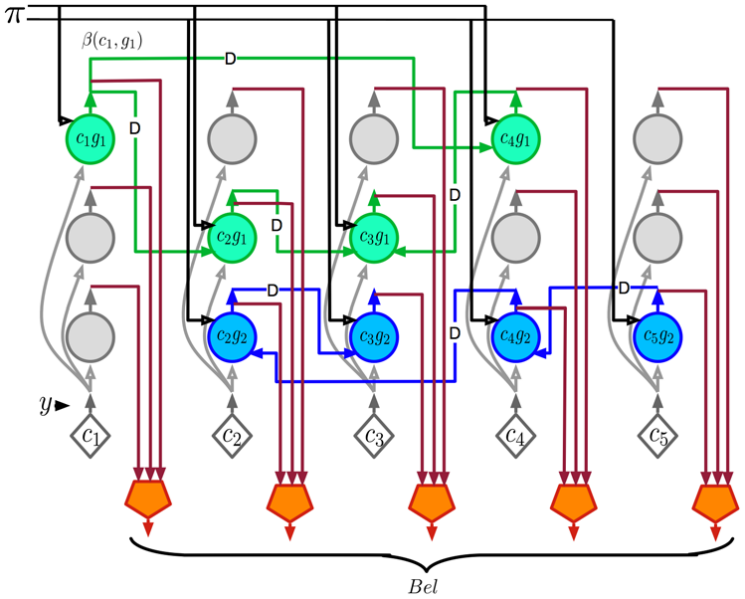
Belief circuit. Circuit for calculating the belief distribution over coincidence patterns by integrating the sequence of bottom-up inputs with the top-down inputs. The pentagon-shaped neurons are the belief neurons. These neurons pool over all the neurons representing the same coincidence in different Markov chains to calculate the belief value for each coincidence pattern. This circuit implements the Equation 6 in Table 1.

The belief calculation, described in Equation 6, has almost the same form as the forward dynamic programming Equations 4. The state variable 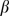 has the same form as the state variable 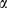 and a very similar update equation. The only difference between these two is the multiplication by a top-down factor 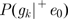 in the belief calculation equations. Therefore, the neuronal implementation of the dynamic programming part of the belief calculation equation is very similar to that of the forward dynamic programming variable 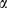. This implementation is shown in Figure 5. The filled-circle neurons correspond to the circle neurons in the forward calculation. Note that, in contrast to the circle neurons in Figure 4, the filled-circle neurons now also have a top-down multiplicative input that corresponds to 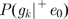.

The pentagon neurons in Figure 5 are the belief neurons. These neurons pool over the activities of the same coincidence neurons in different Markov chains to calculate the belief value for each coincidence pattern. This operation corresponds to the 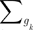 operation in Equation 6. Note that the operation of the pentagon neuron is different from that of the rectangle neuron in Figure 4. The rectangle neuron pools over different coincidence patterns in the same Markov chain. The pentagon neuron pools over the same coincidence pattern in different Markov chains.

#### Calculating the messages to be sent to child nodes

The step that remains to be explained is the conversion of the belief messages to the messages that a node sends to its children. This step is described by Equation 9 and corresponds to the operations performed by the rectangle marked (4) in Figure 2(B). The input for this operation is the belief vector. The outputs are the 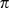 messages that are sent to the child nodes. A message is sent to each child and the message describes the degree of certainty this node has about the child nodes' Markov chains.

**Figure 6:**
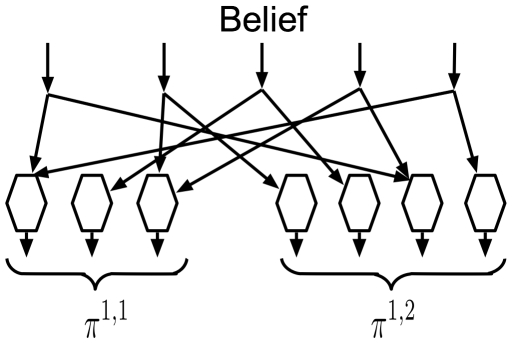
Feedback message circuit. The circuit for computing the messages to be sent to children according to Equation 9. The two sets of hexagonal neurons correspond to the Markov chains of the two children of the reference node.

Figure 6 shows how this equation can be implemented using neurons. The input belief is fed to ‘hexagonal neurons’ that compute the messages for child nodes. Figure 6 shows two sets of hexagonal neurons corresponding to the two child nodes of this node. Each hexagonal neuron corresponds to a Markov chain of the child node. The left child node has 3 Markov chains and the right child node has 4 Markov chains. The outputs of these hexagonal neurons are the messages that are sent to the respective children.

The connections between the input and the hexagonal neurons encode the constituents of coincidence patterns. For example, the first input is connected to the hexagonal neuron representing the first Markov chain of the left child and to the hexagonal neuron representing the third Markov chain of the right child. This is because the coincidence pattern 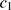 is defined as the co-occurrence of the first Markov chain from the left child and the third Markov chain from the right child. The hexagonal neurons calculate their outputs as a sum of their inputs as described in Equations 9 and 10.

The operation of the hexagonal neurons shown in Figure 6 can be thought of as the reverse of the operations performed by the diamond neurons that were described in Figure 3. The weights on the inputs to both these kinds of neurons define the coincidence patterns. In the case of the diamond neurons, they calculate the probability over coincidences from the probability distribution over Markov chains from each child. The hexagonal neurons do the reverse; they calculate the probability distributions over the Markov chains from each child from the probability distribution over coincidence patterns.

### Further considerations of belief propagation equations

The equations in Table 1 are self-consistent and sufficient for some learning and inference tasks. However, they do not address several issues required for many real world problems. Specifically, they do not address how feedback from a parent node to a child node can influence the child node's feed-forward output, and they do not address issues of specific timing. The following sections address these issues.

#### Role of feedback in the current model

Even though feedback propagation in the current model does not affect feed-forward propagation, it plays an important role in understanding the evidence presented to a network. For example, for an image given as input to the network, feed-forward propagation results in a distribution at the top level about the objects that could be present in the image. Feedback propagation can then be used to identify the features in the input image that produced a particular hypothesis at the top, to identify whether a particular edge in the image belongs to an object or to the background, and to assign ownership of features if there are multiple objects in the scene. In the Results section we give examples of feedback propagation in the current model.

#### Role of feedback in loopy graphs

In the general case, nodes in an HTM network will have overlapping receptive fields. This gives rise to HTM network structures where each node in the network can have multiple parents. Such network structures are ‘loopy’ because of the cycles in their underlying graphs. Belief propagation is theoretically guaranteed to give accurate results in non-loopy graphs. Even though theoretical guarantees do not exist for belief propagation in loopy graphs, it is found to work well in practice on many problems involving loops [32],[33].

HTM nodes with multiple parents can be treated like the causal poly-tree structures described by Pearl [29]. Poly-tree structures imply that multiple higher-level causes influence a lower level cause. Belief propagation computations in poly tree structures have the property that the message from a child to a parent is influenced by the messages from all other parents to the child. This modifies the flow of information in the HTM node in Figure 2(b) by introducing an arrow between box 3 and box 2. Local poly-tree structures can produce loops if repeated in a hierarchy. These sources of loops are likely to be common in brains. Multiple top-down causes can be combined efficiently using the noisy OR-gate structures described in Pearl's book [29].

For the sake of simplicity of exposition, we deal exclusively with singly connected (non-loopy) networks in this paper. It is straightforward to extend belief propagation in HTMs to include multiple parents for each node.

#### Role of feedback for attention

The propagation equations in Table 1 compute approximate posterior distributions at every node in the hierarchy given the evidence. Calculating the posterior distributions at every node is one type of query that can be answered using such propagation techniques. Making inferences about real world situations often requires more complex queries involving a subset of nodes. Pearl discussed this need in his book as a motivation for controlling attention [29] (page 319), “In many cases, there is a small set of hypotheses that stand at the center of one's concerns, so the reasoning task can focus on a narrow subset of variables, and propagation through the entire network is unnecessary.”

For example, one interesting query to answer is what would be the evidence in support of top-level hypothesis 1, if all other top-level hypotheses are assumed to be inactive. This is a form of top-down attention. It can be achieved using local computations by propagating down the influence of hypothesis 1, and inhibiting, at all levels, the bottom-up support that conflicts with the high level hypothesis. In the case of vision, such a mechanism can be used to pay attention to a single object when multiple objects are present in the scene. The top-down propagation of a higher-level hypothesis will, in this case, identify the lower level nodes and coincidence patterns that support the hypothesized object. Turning off all other nodes can increase or decrease the certainty in that hypothesis.

Another form of query that can be answered is to ask what other hypotheses might be active if the top-level hypothesis is considered to be inactive. For example, while recognizing a complex scene, it could be advantageous to not pay attention to an object that is already recognized so as to focus on other objects in the scene. This requires a mechanism that propagates down the currently active hypothesis and turning off all the evidence that supports this hypothesis exclusively.

Both of the above cases correspond to gating the bottom-up evidence using top-down activation. The gating signal at each node, corresponding to an activated top-level hypothesis, can be derived from the computed beliefs at that node. However, maintaining this gating during further computations requires external control mechanisms that are not part of the standard belief propagation machinery. There are several places where this gating can be applied, at the inputs to coincidences, at the coincidences themselves, or at the output of the Markov chains.

#### Incorporating variable speed and duration into the belief calculation

As expressed in the equations, the Markov chains advance their state with every time tick and can model only sequences that happen at a particular speed. The prime enabler of sequential inference in those equations is the property that the outputs of the pre-synaptic neurons at time 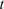 is available at the lateral input of the post-synaptic neuron at time 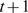, exactly at the time when the bottom-up activity of the post-synaptic neuron arrives. If this lateral activity is maintained at the lateral input of the post-synaptic neuron for a longer duration, the bottom-up input activity for the post synaptic cell do not need to arrive exactly at time 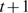. The lateral input that is maintained in the post-synaptic cell can be extinguished when either sufficient bottom-up activity arrives at the post-synaptic cell to produce a ‘firing’ event or when a temporal window is exhausted after the pre-synaptic event. Such a mechanism that strengthens the correct sequence as long as the next event arrives within a temporal window after the previous event would enable variable speed sequential inference that is robust to local temporal warps. Achieving this in the equations requires writing the dynamic programming equations using events rather than time steps, where events are defined using thresholds on the combined lateral and bottom-up activity. Variable speed inference can be achieved with the same neuronal connectivity we showed for fixed speed inference if we assume that mechanisms for maintaining lateral activity and for determining event thresholds are implemented within each neuron. Further explication of this mechanism is left for future work.

Another element missing from the equations in the previous section is an explicit duration model associated with the states of Markov chains. In certain cases of temporal inference, the next event is expected at a precise time after the previous event rather than in a temporal window as discussed in the above paragraph. Music is one example. Humans also have the ability to speed up and slow down this specific duration mechanism. Several techniques exist for incorporating explicit duration models into Markov chains [34],[35]. Some of these techniques introduce self-loops into the Markov chain states. However, self-loops lead to an exponential duration probability density that is inappropriate for most physical signals [35]. Instead, we assume that durations are signaled to a node by an external timing unit that determines the rate of change of the signals using some system-level measurements. This means that the state change computations will have two components. The first component, as described in the previous sections, determines the distribution of the next state without considering when exactly that distribution is going to be active. The second component, the external timing signal, determines when the belief distribution is going to be active.

**Figure 7:**
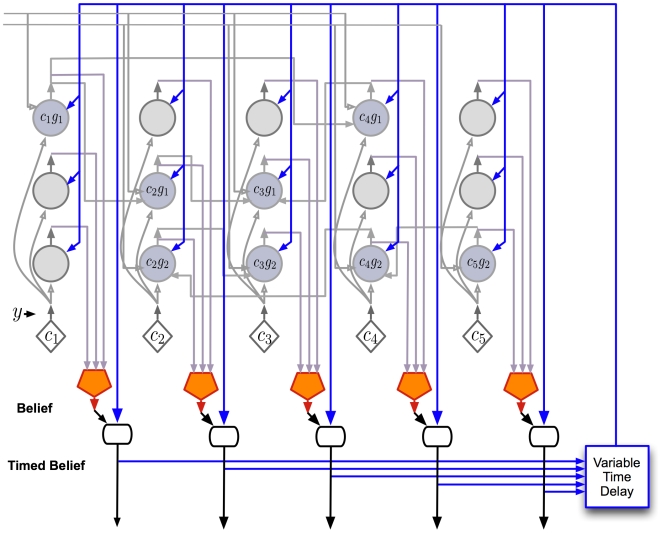
Timing circuit. The same circuit as shown in Figure 5 with the addition of circuitry for incorporating variable time delays between elements of the Markov chains. The pentagon neurons represent the belief at each node. The rounded rectangle neurons represent the belief at each node at the appropriate time delay. An external variable time delay mechanism provides time duration information to all the neurons involved in encoding sequences.

Figure 7 is similar to Figure 5 with the addition of a variable time-delay mechanism. Two types of belief neurons are shown. The pentagonal neurons, previously shown in Figure 5, calculate the node's belief, and rounded rectangle neurons represent the belief at a particular time delay. The outputs of the rounded rectangle neurons are passed through an external variable delay unit. The rounded rectangle neurons act as a gate that opens only when a timing signal and a belief value are both available at its inputs. The activation of these neurons triggers the next timing cycle. The timing signal is used to gate the 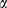 and 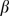 calculations. Only the gating of 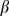 calculation is shown in the figure. The external timing circuit achieves the effect of a specific duration model whose tempo can be changed.

## Results

In this section, we interpret anatomical data of the neocortex within the context of the computational specifications from the previous sections. Anatomical data gives us important constraints on input and output layers, intra- and inter-laminar connections and placement of cell bodies and dendrites. Assignment of a particular function to a particular layer imposes constraints on what functions can be performed by other layers. The challenge is to find an organization that is self-consistent in the sense that it implements the belief propagation equations while conforming to the constraints imposed by biology.

Our working hypothesis can be stated simply: The cortical circuits implement the HTM belief propagation equations described in Table 1. A hypothetical neuronal implementation of these equations was described in the previous section. Under the assumption that the cortical circuits are implementing these equations, what remains to be explained is how the abstract neuronal implementation of the previous section is physically organized in the layers and columns of actual cortical anatomy. This is accomplished by comparing the abstract neural implementations with anatomical data. We describe the results in two stages. First we describe the high-level mapping between the neocortical hierarchy and the HTM hierarchy. Then we describe how the circuits based on HTM belief propagation equations can be mapped to cortical columns and laminae.

### Mapping between neocortex hierarchy and HTM network hierarchy

An area of cortex can be thought of as encoding a set of patterns and sequences in relation to the patterns and sequences in regions hierarchically above and below it. The patterns correspond to the coincidence patterns in an HTM node and the sequences correspond to the Markov chains.

**Figure 8:**
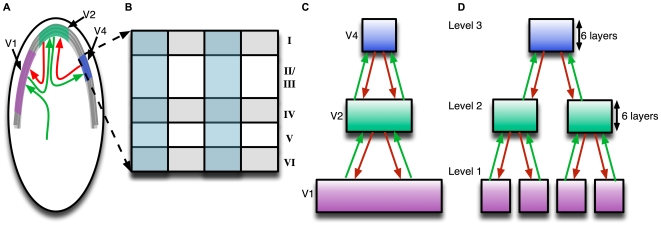
Mapping between neocortex hierarchy and HTM hierarchy. (A) Schematic of neocortex inside the skull. The neocortex is a thin sheet of several layers of neurons. Different areas of the neocortical sheet process different information. Three successive areas of the visual hierarchy – V1, V2 and V4 – are marked on this sheet. The connections between the areas are reciprocal. The feed-forward connections are represented using green arrows and the feedback connections are represented using red arrows. (B) A slice of the neocortical sheet, showing its six layers and columnar organization. The cortical layers are numbered 1 to 6: layer 1 is closest to the skull, and layer 6 is the inner layer, closest to the white matter. (C) Areas in the neocortex are connected in a hierarchical manner. This diagram shows the logical hierarchical arrangement of the areas which are physically organized as shown in (A). (D) An HTM network that corresponds to the logical cortical hierarchy shown in (C). The number of nodes shown at each level in the HTM hierarchy is greatly reduced for clarity. Also, in real HTM networks the receptive fields of the nodes overlap. Here they are shown non-overlapping for clarity.

An HTM Node, as described earlier in this paper, encodes a set of mutually exclusive patterns and Markov chains. A region of cortex that has several patterns simultaneously active will be implemented using several HTM nodes. Figure 8(D) shows the HTM implementation of the logical cortical hierarchy shown in 8(C). This arrangement corresponds to one of the basic organizing principles of the visual system where neurons in higher-level visual areas receive inputs from many neurons with smaller receptive fields in lower-level visual areas [36]. In addition, due to the temporal nature of HTM, this arrangement corresponds to a temporal hierarchy analogous to the kind reported by Hasson and colleagues [37]. In this highly simplified mapping, the area V1 is implemented using 4 HTM nodes while area V2 is implemented using 2 HTM nodes. Typically, the number of non-exclusive patterns that needs to be maintained decreases as you ascend in the hierarchy. Therefore, higher-level cortical regions can possibly be modeled using a fewer number of HTM nodes. Note that this is a representative diagram. A cortex-equivalent implementation of V1 and V2 could require several thousand HTM nodes for each cortical area and the receptive fields of the nodes would typically be overlapping.

The coincidence patterns and Markov chains in an HTM node can be represented using random variables. A cortical column can be thought of as encoding a particular value of the random variable that represents the coincidence patterns in the HTM node. The feed-forward and feedback connections to a set of cortical columns carry the belief propagation messages. Observed information anywhere in the cortex is propagated to other regions through these messages and can alter the probability values associated with the hypotheses maintained by other cortical columns. In HTMs these messages are computed using the mathematics of Bayesian belief propagation as we described earlier.

### A detailed proposal for the computations performed by cortical layers

**Figure 9:**
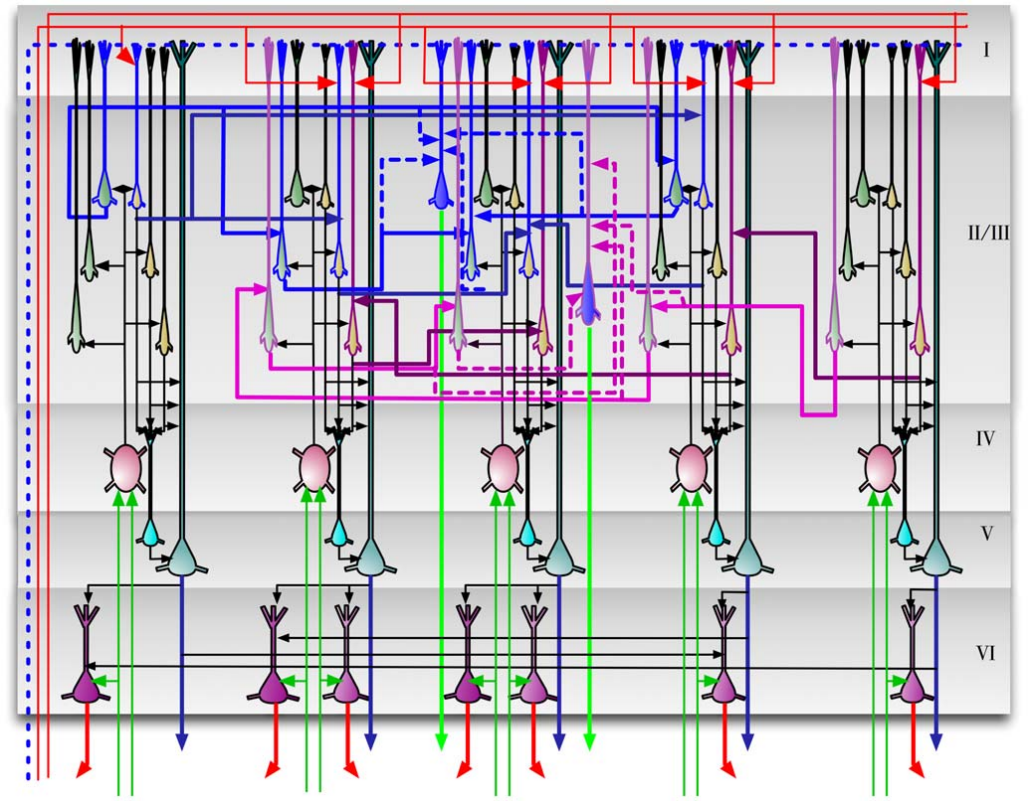
A laminar biological instantiation of the Bayesian belief propagation equations used in the HTM nodes. The circuit shown here corresponds exactly to the instantiation of the reference HTM node shown in Figure 2. The five vertical ‘columns’ in the circuit correspond to the 5 coincidence patterns stored in the reference node. Layers 1 to 6 are marked according to the standard practice in neuroscience. Emphasis is given to the functional connectivity between neurons and the placement of the cell bodies and dendrites. Detailed dendritic morphologies are not shown. Axons are shown using arrow-tipped lines. Feed-forward inputs and outputs are shown using green axons and feedback inputs and outputs are shown using red axons. Whether an axon is an input or output can be determined by looking at the direction of the arrows. The blue axons entering and exiting the region represent timing-duration signals. ‘T’ junctions represent the branching of axons. However, axonal crossings at ‘X’ junctions do not connect to each other. Inter-columnar connections exist mostly between neurons in layer 2/3, between layer 5 cells, and between layer 6 cells. The inter-columnar connections in layer 2/3 that represent sequence memories are represented using thicker lines.

Our proposal for the function, connectivity and physical organization of cortical layers and columns is shown in Figure 9. This figure corresponds to the laminar and columnar cortical circuit implementation of the belief propagation equations for the reference HTM node in Figure 2. Figure 9 was created by arranging the neurons of the abstract neuronal implementation of HTM belief propagation into columns and laminae in such a way that the resultant circuit matched most of the prominent features found in mammalian neocortex. In the following sections we de-construct this picture and examine the anatomical and physiological evidences for the specific proposals. This will also illuminate the process that we went through to arrive at the circuit shown in Figure 9.

The circuits in Figure 9 provide an exemplar instantiation of the Bayesian computations in laminar and columnar biological cortical circuits. Several plausible variations and exceptions of this circuit can be found because of the degrees of freedom in the implementation of the belief propagation equations and because of the incompleteness of anatomical data. We will tackle some of these exceptions and variations as they come up in the appropriate context and also in the Discussion section.

#### Columnar organization

The cortical circuit shown in Figure 9 is organized as 5 columns corresponding to the 5 coincidence patterns in the reference HTM node that we started with. The neurons in each column represent some aspect of the coincidence pattern that the column represents. For example, the neurons in layer 2/3 represent the coincidence pattern in the context of different sequences, whereas the neurons in layer 6 represent the participation of the coincidence pattern in the calculation of feedback messages. The 5 columnar structures shown represent a set of 5 mutually exclusive hypotheses about the same input space. For example, these columns can correspond to a set of columns in the primary visual cortex V1 that receive input from a small area of the visual field. The 5 coincidence patterns might correspond to different orientations of a line. If the receptive field is small enough, the different orientations can be considered mutually exclusive - the activity of one reduces the activity of the other. This kind of columnar organization is typical in biology [38],[39].

In the idealized cortical column model, each different aspect that needs to be represented for a coincidence pattern is represented using a single neuron. For example, there is exactly one neuron representing coincidence pattern 1 in the context of Markov chain 1. This means that there is no redundancy in this idealized cortical representation. Nothing about the computation or the representation changes if we replicate each neuron in this circuit a few times, while maintaining their connectivity. A coincidence that is represented by a single neuron in our cortical column can be represented by a cluster of laterally interconnected neurons.

**Figure 10:**
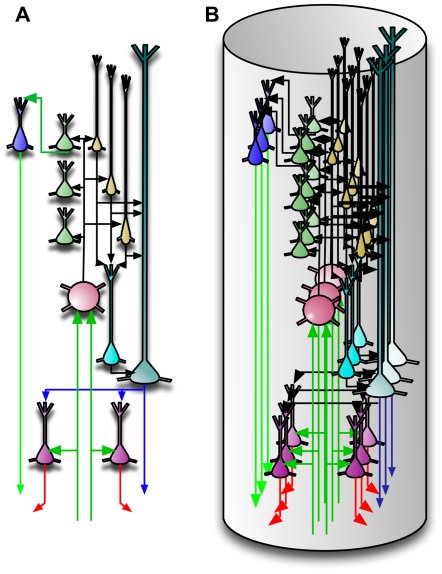
Columnar organization of the microcircuit. (A) A single idealized cortical column. This idealization could correspond to what is often referred to as a biological mini-column. It is analogous to one of the five columnar structures in Figure 9. (B) A more dense arrangement of cells comprising several copies of the column (A). Although we typically show single cells performing computations, we assume there is always redundancy and that multiple cells within each layer are performing similar functions.

One prediction of our model is that many of the connections within a vertical column of cells can be established without any learning. Figure 10(A) shows a single idealized column. The connections within this column that can be established a-priori are shown in black. These connections act as a backbone for carrying out belief propagation computations. This feature makes our idealized cortical column a good candidate to be a developmental feature. The idealized cortical column in Figure 10(A) can correspond to what is known as the *mini-columns* or *ontogenetic columns* of the cortex [40]. Mini-columns are developmental units that contain about 80 to 100 neurons. By the 26th gestational week, the human neocortex is composed of a large number of mini-columns in parallel vertical arrays [41]. In real brains we would not want to represent something with a single cell. Therefore, we assume that in real brains the basic computational column will consist of many redundant cells bound together using common input and short-range intra-laminar connections resulting in a column as shown in Figure 10(B) [41].

For the rest of the discussion we will focus on the idealized cortical column and the idealized cortical circuit with no redundancy.

#### Layer 4 stellate neurons implement the feed-forward probability calculation over coincidence patterns

The excitatory neurons in layer 4 of the cortex consist primarily of star-shaped neurons called stellate neurons and pyramidal neurons [42]. Layer 4 is generally accepted as the primary feed-forward input layer to cortical regions [2]. In the cat primary visual cortex, the outputs from the retina pass through the lateral geniculate nucleus (LGN) of the thalamus and then terminate on layer 4 stellate cells. Most of these connections are known to be proximal to the cell body and can drive the cells. The major projection (output) of layer 4 stellate neurons is to layer 3 cells [15].

We propose that the layer 4 stellate cells implement the probability calculation described in Equation 2 and shown in Figure 3. This means that layer 4 neurons are coincidence detectors and that the synapses of the layer 4 neurons represent co-occurrence patterns on its inputs.

We realize this is a dramatic simplification of layer 4 cell connectivity. It does not address the very large number of synapses formed on distal dendrites, nor does it address the fact that many feed-forward connections from Thalamus terminate in layer 3 cells and that in some regions of cortex layer 4 is greatly diminished. These facts can be supported by HTM theory. The horizontal connections between layer 4 cells can implement spatial pooling or temporal pooling without timing. Layer 3 cells can also act as coincidence detectors of inputs from thalamus that make proximal connections, and layer 3 cells can take the full burden of coincidence detection. However, we choose to present the simplest explanation of layer 4 cells for clarity and discuss some of the variations in the Discussion section.

In Figure 9, the layer 4 neurons are shown in red. The inputs to these neurons are the outputs of lower levels of the cortical hierarchy, possibly routed through the thalamus. It is easy to verify that the connections of these neurons correspond to the ‘diamond’ neurons in our belief propagation implementation shown in Figures 3 , 4 and 5. Note that in the implementation of the belief propagation equations shown in Figures 4 and 5, the neurons that calculate the probability distribution on coincidence patterns (the diamond neurons) have only feed-forward inputs. This is in contrast to many other neurons that receive feed-forward, feedback and lateral inputs. In neuroscience, it is accepted that the feedback inputs to a cortical region generally avoid layer 4 [2]. This is consistent with our proposal for the function of layer 4 neurons.

Making layer 4 correspond to the feed-forward computation of the probability over coincidence patterns imposes some constraints on the computational roles for other layers. For example, the major projection of layer 4 is to layer 3. This means that any computation that requires major inputs from layer 4 will need to be done at layer 3 and should match the general characteristics of layer 3. The proposals for layer 3 computations, described in a subsequent section, match these constraints.

#### Layer 1: The broadcast layer for feedback information and timing information

Feedback connections from higher levels of the cortex rise to layer 1. The recipients of these connections are the cells with apical dendrites in layer 1. Layer 1 is comprised mostly of axons carrying feedback from higher levels of cortex, axons from non-specific thalamic cells, apical dendrites, and a minor concentration of cell bodies [42].

To remain consistent with this biological data, the layer 1 in our mapping will be the ‘broadcast’ layer for feedback and timing information. The axons carrying feedback information 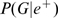 will be available at layer 1 and accessed by the apical dendrites of neurons that require this information. In addition, the availability of a timing signal at layer 1 is assumed. The purpose of this timing signal is discussed in a subsequent section where we discuss the layer 5 cells.

#### Layer 2/3 pyramidal cells: Sequence memory, pooling over sequences, incorporating feedback information

The primary inter-laminar excitatory input to layer 2/3 is from the stellate cells of layer 4. In addition, the layer 2/3 neurons receive excitatory inputs from other layer 2/3 neurons via extensive lateral connections [43]. Many layer 2/3 neurons project to higher levels of cortex and to layer 5 [44].

We propose three different roles for the layer 2/3 pyramidal neurons in cortical circuits: (1) Calculation of feed-forward Markov chain (sequence) states, (2) Projection of Markov chain information to higher level cortical areas, and (3) Computation of sequence states that incorporate feedback information. We now consider each proposal in detail and then examine anatomical evidence in support of these circuits.

1. **Pyramidal cells for calculating feed-forward sequence states:** The pyramidal neurons shown in green in Figure 9 implement the Markov chain sequences and the dynamic programming computations for feed-forward sequential inference. These neurons correspond to the ‘circle neurons’ that we described in the Model section and implement the dynamic programming Equation 4 in Table 1. These pyramidal neurons receive ‘vertical’ excitatory inputs from the outputs of layer 4 stellate neurons and ‘lateral’ inputs from other pyramidal cells within layer 2/3. Circuits in layer 2/3 of Figure 9 show our proposal for how Markov chain sequences are implemented in biology. The green pyramidal neurons with blue outlines and blue axons correspond to Markov chain 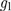 in Figure 4 and the green pyramidal neurons with magenta outlines correspond to Markov chain 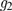 in Figure 4. The axons from these pyramidal cells cross column boundaries and connect to other pyramidal neurons that belong to the same sequence. Since these connections correspond to learned sequence memories, they will be very precise about which columns and which specific neurons within these columns they target.
2. **Pyramidal cells that project to higher order cortex:** The next issue we want to address is how the Markov chain identities are sent to higher level cortical regions. We see several possibilities. One is to use a second set of pyramidal cells in layer 2/3. These pyramidal cells correspond to the Markov chain identities and get excitatory inputs from the layer 2/3 pyramidal cells that belong to the same Markov chain. This second set of pyramidal neurons in layer 2/3 corresponds to the rectangle neurons in Figure 4. These neurons send their outputs to higher-level cortical regions. In Figure 9, these pyramidal neurons are shown in blue color in layer 2/3 and send their axons down to the white matter to reach higher cortical areas. The second proposal does not require a second set of neurons but instead relies on long lasting metabotropic responses of neurons. The cells in layer 3 which represent the individual elements in Markov chains will become active in turn as sequences are learned and recalled. We need a way of generating a constant response that persists as the individual sequence elements are traversed. If the layer 3 cells that represent the sequence elements project to metabotropic receptors in higher cortical regions, those destination neurons could stay active for the duration of sequences. Strong evidence suggesting which of these two, or other, mechanisms is used is lacking. It is a strong theoretical prediction that a mechanism must exist in each region of cortex for forming constant representations for sequences of activity. It is an area for further study to determine what is the most likely mechanism for this.
3. **Pyramidal cells for computing sequences based on feedback:** In the Model section, we saw that a second set of dynamic programming states were required for the calculation of the belief of coincidence patterns and as an intermediate step in deriving the feedback messages to be sent to the children. These neurons do the sequence computations while integrating feedback information from the higher layers. We propose a third set of pyramidal neurons in layer 2/3 for this purpose. These neurons correspond to the filled-circle neurons in Figure 5. In Figure 9, these neurons are represented using yellow colored pyramidal neurons in layer 2/3. The lateral connections of these neurons are similar to the lateral connections of the layer 2/3 green pyramids that we just described. However, these yellow layer 2/3 neurons also integrate feedback information from layer 1 using their apical dendrites in layer 1 as shown in Figure 9. A prediction arising from this mechanism is that two classes of neurons in layer 2/3 can be differentiated by the connections they make in layer 1. One class of layer 2/3 neuron will form layer 1 synapses with feedback axons from higher levels of cortex. The other class of layer 2/3 neuron will not form synapses with feedback axons, but will form synapses with axons from non-specific thalamic cells needed for timing (discussed more in a later section).

Now, let us examine the anatomical evidence that led us to these proposals. The major bottom-up input required for the above calculations is the feed-forward probability over coincidence patterns that was assigned to layer 4 neurons in a previous section. The major excitatory projection of layer 4 neurons is to layer 2/3 neurons [45]. For example, L4 spiny neurons in the barrel cortex of the mouse are characterized by mainly vertically-oriented, predominantly intra-columnar, axons that target layer 2/3 pyramidal cells [46]. Note that the green and yellow neurons in Figure 9 receive inputs from layer 4 neurons that are in the same column.

Cells in layer 2/3 are known to be ‘complex’ cells that respond to sequence of motion or cells that respond invariantly to different translations of the same feature. Unlike cells in layer 4 that respond to more impoverished stimuli, cells in layer 2/3 of the visual and barrel cortices strongly prefer richer stimuli, such as motion in the preferred direction [47]. This is consistent with our proposal that most layer 2/3 cells represent different coincidence patterns in the context of different Markov chain sequences. They become most active only in the context of the correct sequence. In biology, it is found that axons of the layer 2/3 pyramidal neurons travel several millimeters parallel to the layer 2/3 – layer 4 boundary and re-enter layer 2/3 to make excitatory connections to pyramidal cells there [43],[48]. This is akin to the blue and magenta axons that we show in Figure 9 and is consistent with the implementation of sequence memories and dynamic programming computations. The green neurons and the yellow neurons in Figure 9 correspond to this description and are assumed to encode states within sequences.

We show green and yellow layer 2/3 neurons in Figure 9 because we need to learn two sets of sequences. One set of sequences is used in feed-forward calculations and the other set of sequences is used in feedback calculations. In our figures the green neurons are feed-forward and the yellow neurons feedback. The yellow neurons need to have apical dendrites in layer 1 to receive feedback from higher cortical areas. The green neurons may also have apical dendrites in layer 1 to receive timing information. But the green feed-forward neurons should not make connections with the feedback signal. This is a theoretical prediction currently without experimental data for support or falsification.

The computation that the sequence state cells in layer 2/3 need to perform for inference involves a weighted sum of their lateral connections multiplied by a bottom-up input. We found several data points suggesting that neurons in layer 2/3 are capable of approximating a similar computation. Yoshimura et al [49] report that long distance horizontal connections to pyramidal cells in layer 2/3 exhibit different properties than those from vertical connections. They found that, under depolarized conditions, the EPSP evoked by the activation of an individual input pathway (either horizontal or vertical, but not both) was smaller than that evoked without the depolarization. They also found that when both the vertical and horizontal inputs were driven simultaneously, the evoked EPSP was larger than the mathematical summation of the individual EPSPs. They concluded that this indicated multiplicative supralinear summation of EPSPs caused by simultaneous activation of long range horizontal and vertical inputs under depolarized conditions, and suggested that the observed nonlinear summation is attributable to the intrinsic membrane properties of the pyramidal cells or the synaptic properties of the inputs, rather than the properties of the global neuronal circuitry. Another study [50] suggested that the projections of layer 4 spiny neurons to layer 2/3 pyramidal neurons act as a gate for the lateral spread of excitation in layer 2/3.

Our model requires that sequences at higher levels of the hierarchy represent longer durations of time. The difference in temporal scales can be orders of magnitude depending on the depth of the hierarchy. In the Model section, we outlined how variable durations can be encoded in the same sequence circuit by maintaining lateral inputs to the post-synaptic neurons for a temporal window. The biological mechanisms underlying such maintained activity is not well understood. One possibility is that these activities are mediated by pre-synaptic calcium [51]. The layer 2/3 circuit that we described can be thought of as a minimal set of circuits that are needed for temporal inference on multiple scales. If intrinsic properties of neurons are not adequate to represent the longer time scales required by our model, it can be achieved via additional network mechanisms. A network mechanism to this effect is described in a subsequent section.

To calculate the belief in a coincidence pattern, the outputs of all the yellow neurons in the same column have to be summed up. This corresponds to pooling the evidence for that coincidence pattern from all the different Markov chains (sequences) in which the coincidence participates. Layer 5 is ideally suited for doing this. It is known that layer 2/3 pyramidal cell axons have two distinct projection fields: one horizontal (the long range axon collaterals), and one vertical [46]. The horizontal, trans-columnar connections target other layer 2/3 pyramidal cells [52],[53] and correspond to the sequence memory circuits that were described above. Both the green neurons and the yellow neurons in Figure 9 take part in these circuits, with the yellow neurons receiving feedback information as well. It is known that the trans-laminar projections of layer 2/3 neurons are to a class of cells known as layer 5-B [44]. It is also known that layer 3 pyramidal cells that connect to layer 5 cells have their apical dendrites in layer 1. The projections from layer 3 to layer 5 are confined to the same column [46]. In the next section we will see that this is consistent with our proposal for the belief calculation cells in layer 5.

#### Layer 5: Implementation of belief calculation

We propose that a class of layer 5 pyramidal neurons in cortical circuits calculate the belief over coincidence patterns according to Equation 6. This corresponds to the computations performed by the pentagonal neurons in Figure 5. In the biological implementation shown in Figure 9, these neurons are shown in light cyan color in layer 5. These neurons receive inputs from the yellow neurons in layer 2/3. Logically, the operation of these layer 5 belief neurons corresponds to the pooling of evidence for a particular coincidence from the different sequences that this coincidence participates in.

#### Layer 5 pyramidal cells for duration models

As mentioned in the Model section, a method of encoding time duration is needed in memorizing and recalling sequences within the Markov chains. Exactly how this is done is not critical to the main ideas in this paper. However, the biological possibilities for encoding duration are somewhat limited and one possible implementation suggests itself. In this section we explore this mechanism starting with some assumptions that led to it.

Our model makes the assumption that cortical circuits store duration of individual elements within sequences and that the mechanism used to store duration must be present in many if not all cortical areas. Further, a human can store specific durations, such as duration of notes in music, that last up to about a second. This is too long for typical process delay times in neurons, suggesting the existence of a separate duration encoding mechanism. Humans also have the ability to speed up and slow down memorized sequences during recall, which suggests a partially centralized mechanism that can influence the rate of recall over multiple elements in a sequence. Duration information must also be available over broad areas of cortex so that duration information can be associated between any subsequent elements in a Markov chain. And finally, encoding duration between elements in a sequence requires a signal that marks when a new element has started. This suggests the need for cells with a brief burst of activity. When we looked for anatomical data that satisfied these constraints, the cortical projections to and from non-specific thalamic cells were the best fit.

In the proposed circuit, layer 5 pyramidal cells remember the precise time at which a belief is going to be active as measured as a duration from the previous element in the sequence. These neurons, shown as the dark cyan neurons in the layer 5 of Figure 9, correspond to the rounded-rectangle neurons in Figure 7. The timing signal, assumed to be broadly available in layer 1, is shown as blue colored axons. The dark cyan timing neurons have their apical dendrites in layer 1 to access this timing signal. It is assumed that the belief-timing neurons project to non-specific thalamic regions (the thalamic matrix) [54] which implement a variable delay mechanism that projects back to layer 1 to complete a timing loop, as shown in Figure 7. LaBerge's [55] research has identified the recurrent connection from layer-5 to the matrix thalamus to the apical dendrites of layer 2/3 and layer 5 neurons as the circuit responsible for sustaining activity for extended durations to support cue-target delay tasks. The connections through the matrix thalamus have also been proposed as a mechanism for thalamo-cortical synchrony [54].

Now let us examine the anatomical evidence for these neurons and connections. There are primarily two kinds of pyramidal neurons in layer 5 of the cortex. The first type are called ‘regular-spiking’ (RS) neurons and the second type are called ‘intrinsically bursting’ (IB) neurons. The IB cells are larger, they extend apical dendrites into layer 1, and as their name suggests they exhibit a burst of action potentials when they become active. The RS cells are smaller, their apical dendrites are mostly in superficial layer 4, and they exhibit a stream of action potentials when active. It is also known that the RS cells are mostly pre-synaptic to the IB cells [43]. That is, RS cells send their outputs to IB cells. In our mapping in Figure 9, the RS cells are the light-cyan colored neurons in layer 5. The IB cells are the dark-cyan colored neurons in layer 5 with their apical dendrites in layer 1. The output of the RS cell goes to the IB cell. These mappings are consistent with anatomical data [42],[45].

Most of the excitatory connections from the layer 2/3 pyramidal cells (the yellow neurons in Figure 9) to layer 5 go to the IB cells [42]. This layer 2/3 input, plus the apical dendrite extending to layer 1, and the bursting behavior, suggest the IB cells are ideally situated for both the pooling of evidence and encoding the beginning (and hence timing) of a sequence element.

What role then might the RS cells play? In our survey, we could not find detailed information about the inputs to RS cells. The existence of RS cells can be justified if there is utility in representing a belief in a coincidence pattern that does not incorporate precise timing information. Introspection leads us to believe that there is indeed the need for such a neuron. Consider the case of listening to music. We anticipate which note is going to happen next, well before it happens. The RS cells in layer 5 can be thought of as belief cells that ‘anticipate’ the occurrence of the belief, whereas the IB cells represent the same belief at a precise time point.

The RS cells are known to project to sub-cortical areas like the striatum and the superior colliculus [42] where the anticipation signal could be used to anticipate actions. The IB neurons of layer 5 also project to sub-cortical areas and to motor areas. If a cortical area is to influence actions, it makes sense that the signals for that should be based on the belief of that cortical area, because the belief represents the best possible information about the coincidence patterns represented in that cortical area. Therefore, the fact that layer 5 RS neurons and IB neurons project to sub-cortical areas that influence motor actions is consistent with the proposal that they compute the belief.

The timing loop requires the projection of the IB neurons to an external timing circuit. Hawkins [7] has proposed the projections of IB cells to the non-specific thalamus as the mechanism for generating a variable timing signal. Non-specific thalamic cells were recommended for this role because they have the required connectivity, receiving input from layer 5 cells and projecting broadly back to layer 1. Beyond this connectivity there is nothing else to support this conjecture. Our literature search and private conversations with several thalamic anatomists have yielded no evidence of anyone ever recording from non-specific thalamic cells. For this conjecture to be true we would expect to see something like a cascade of non-specific cells become active in sequence in response to a burst on a layer 5 IB cell. The cascade would last for several hundred milliseconds.

We can imagine alternate mechanisms for encoding duration. For example, the cerebellum is known to encode specific timing and its connectivity to the thalamus suggests it could play this role. However, the layer 1 synapses of layer 5 IB cells appear to be in a more efficient and logical location for storing duration in that these synapses are directly connected to the beliefs being calculated in layer 5. Again the result of this paper, the mapping of a hierarchical belief propagation model onto cortical anatomy, does not depend on how duration is encoded, but it does require it is encoded by some means that can handle time scales varying in orders of magnitude. LaBerge's research [55] provides a summary of arguments for the hypothesis that a recurrent cortico-thalamo-cortical circuit as proposed here can provide stable levels of modulatory activity at the soma of cortical pyramidal neurons that can persist over extended periods of time. However, it is noted that the biophysical and network mechanisms underlying persistent temporal representations is still an area of active research.

#### Layer 6: Computing the feedback messages for children

We assign to layer 6 pyramidal neurons the role of computing the feedback messages that are to be sent to regions that are hierarchically below. This corresponds to the hexagonal neurons in Figure 6 and Equation 9 in Table 1. In Figure 9, these are shown as the purple colored neurons in layer 6. Feedback messages are derived from the results of the belief calculations from a set of columns. This means that the layer 6 neurons will receive inputs from the layer 5 neurons involved in the calculation of beliefs. This is shown in Figure 9. A given set of columns will send feedback messages to all its ‘child regions’. The feedback message sent to one child is not the same as the feedback message sent to the other child. In Figure 9, some of the layer 6 neurons project to the left child while the rest project to the right child.

Layer 6 is known to be a primary source of cortical feedback connections [15]. There is a class of pyramidal neurons in layer 6 that have short dendritic tufts extending primarily to layer 5. The axons of these neurons project cortico-cortically [42] in a feedback direction. Hence they are appropriately situated for calculating the feedback messages and their connectivity is consistent with our proposals for other layers. Note that in Figure 9, the axonal inputs to layer 6 neurons from layer 5 neurons cross several columns. The input connections to a layer 6 cell come from the columns corresponding to the coincidence patterns that have the child nodes Markov chain as a component.

In Figure 9, different layer 6 neurons project to different child nodes. An alternative implementation is for these neurons to be located in layer 2 of the respective child nodes. This implementation has the advantage that the higher-level node can send the same feedback signal to all the child nodes. In either case, the input connections to these neurons represent the participation of the child node's Markov chain in the higher-level node's coincidence patterns. These connections will need to be learned through the simultaneous activation of the bottom-up outputs from children with top-down outputs from the parent.

There are several other neuron types that are identified in layer 6. We do not attempt to explain the functions of those neurons. However, it is worth mentioning that some of the layer 6 cortical circuits already identified by other neuroscientists as possible candidates for the gating of feedforward activation (control of attention) [56]–[59] are compatible with our model. According to these studies, cells in layer 6 of V1 provide extensive feedback connections to the LGN of the thalamus. These feedback connections target the distal dendrites of relay cells and also contact inhibitory interneurons. The same layer 6 cells also send collateral axons to layer 4 where LGN afferents contact the cortex.

The connections that a set of layer 6 neurons makes to layer 4 and the thalamus are ideally suited for the attention control mechanism that we outlined for the HTM belief propagation. We described how the belief responses generated at every level can be used for gating feed-forward evidence in accordance with a top-level hypothesis. These signals need to be passed through control mechanisms that will maintain the gating while further bottom-up and top-down propagation for the attended to stimuli alter the belief neuron responses. A layer 6 neuron that receives input from layer 5 belief cells is ideally situated for this purpose. Why would layer 6 neurons feed back to layer 4 and also to thalamus? One possible explanation is that each connection provides a different kind of attention modulation. For example, the layer 4 connection could be for attending to the coincidence pattern corresponding to the currently active belief and the thalamus connection could be for attending to every coincidence pattern that is not part of the current belief. These conjectures about layer 6 cells need further research and refinement.

#### Exceptions from model

The six-layered cortical architecture we have described so far is most typical of sensory regions of cortex. Many variations in cortical architecture are known to exist, such as variations in the density and type of cells in a layer, and variations in the number of layers. In addition, there are many known common features of cortical architecture that are not explicitly addressed in our model. Included in this category are the previously mentioned cell types in layer 6 and all the classes of inhibitory cells. These variations and omissions are not necessarily at odds with the model presented in this paper. Not all functions of the belief propagation equations have to be implemented exclusively in one layer. Some layers, such as layer 3 and layer 4 may both be implementing feed-forward coincidence detection and grouping but over differing spatial and temporal resolutions, which could explain why layer 4 typically gets less prominent as you ascend the cortical hierarchy. Different cell types may be needed for short term memory (not included in our model) and different types of attention. Inhibitory cells are needed to implement learning. These topics are beyond the scope of this paper.

Given the behavioral flexibility and resilience of the cortex, we should expect some flexibility in the mapping between a theoretical model and its anatomical instantiation. If our model required a precise and unwavering mapping onto many unique cell types and their connections it is unlikely such a system could evolve. However, we suggest that the mapping of our model to cortical anatomy is sufficiently constrained to suggest its validity and provide testable predictions, but not so constrained to forbid useful variations in its biological implementation. The basic model can remain intact even though variations in timing mechanisms, attention mechanisms, motor mechanisms, etc. are expressed in variations in cortical architecture.

#### Summary

A summary of the proposed computational roles is given in Table 3.

**Table 3:** Summary of anatomical features and their proposed computational functions.

#	Anatomical feature	Proposed computational role
1	Feed-forward thalamic projection to layer 4.	Storage and detection of coincidence patterns.
2	Layer 4 cell dendrites are mostly within layer 4. These cells make vertical projections to layers 2 and 3.	Bottom-up inputs required for the sequence likelihood calculation in equation.
3	Layer 3 cells with inter-columnar lateral projections to other layer 2/3 cells. Some of these cells send their outputs to higher order cortex.	Calculation of sequence likelihoods for feed-forward and feedback calculations.
4	Layer 5 cells with apical dendrites in the superficial layer 4 and bottom of layer 3.	Belief calculation without specific timing.
5	Layer 5 cells with apical dendrites in layer 1. These send outputs to subcortical regions and non-specific thalamus.	Belief calculation with specific timing.
6	Layer 6 neurons with apical dendrites in layer 5.	Computation of feedback messages for child regions.
7	Projections to layer 1 from higher level regions and from non-specific thalamic cells.	High level input is feedback information. Non-specific thalamic input is timing information for Markov chains.

See [42] for details on the anatomical features summarized in this table.

### Object recognition experiments using HTMs

Although the main purpose of this paper is the exposition of HTM theory and its connection to biology, we believe it is useful to discuss our work applying HTMs to practical problems. In this section, we summarize the results of the work being done at Numenta in applying HTMs to the problem of visual object recognition. A detailed treatment of this topic is beyond the scope of this paper.

We started by applying HTMs to a line drawing recognition problem that we call the *Pictures* problem. The Pictures data set consists of line drawings of 48 categories of objects. These line drawings are shown in Figure S1. Each pattern is of size 32 pixels by 32 pixels. The goal was to train an HTM network to recognize test patterns with translations, severe distortions, scale and aspect ratio changes, clutter and noise. The Pictures data set has some properties that make it attractive for applying HTMs. Most objects occupy only a fraction of the 32×32 pixel input. This enables the creation of test images with large translations and scale variations while still maintaining the 32×32 pixel input dimensions. The objects are of different sizes. Some objects (for example, the ‘dog’) contain other objects (the ‘cat’). Most of the objects are constructed from the same set of local features. This means that techniques that use local features alone are not adequate to recognize these objects. The spatial configuration of the local features (i.e, the shape) is important. Recognizing test patterns despite translations, distortions and clutter is a challenging task even on this seemingly simple data set.

We found that HTM network hierarchies with four levels work best for the Pictures task. Adding more levels did not help in improving the recognition accuracy on our test set. The HTM networks are trained in a level-by-level manner, starting with the coincidence patterns and Markov chains at the first level and then moving up the hierarchy. During training, the network is shown programmatically constructed movies in which the objects undergo translations and scale variations in a smooth manner. The training strategy we outlined in the Model section was used for learning the coincidence patterns and Markov chains. More details about the training methods and the learned coincidence patterns and Markov chains can be found in [8]. A representative set of learned Markov chains is shown in Figure S2. A challenging test set was created by programmatically distorting the training images and by adding noise. Examples of test images for the ‘table lamp’ category are shown in Figure S3. The HTM networks reported in our previous work [9] used Markov chains based temporal pooling only at level 1 of the hierarchy and gave 49% recognition accuracy on this test set. We found that incorporating Markov chains based temporal pooling at higher levels increased the recognition accuracy on test sets to 72%. In comparison, a nearest neighbor classifier using exactly the training paradigm used to train the top level of the HTM gives only 35% accuracy. A stand-alone demonstration of this project that lets users interactively draw images to test the network is included with the NuPIC software available for download from Numenta's website (http://www.numenta.com). The network performs impressively in qualitative testing. The Pictures demo, data set, and parameter files are supplied as part of the NuPIC software available from Numenta.

We modified the network structure while maintaining the same spatial and temporal learning/inference algorithms to create an HTM network that can recognize grayscale images. In this network, the first level of coincidences were replaced with Gabor filters of different orientations. At all levels, the coincidence patterns were restricted to have spatial receptive fields smaller than that of the Markov chains. With these modifications, we could successfully train several gray scale image recognition networks. On the standard Caltech-101 benchmark [60], our initial experiments with the network achieved 50% recognition accuracy with 15 training images and 62% recognition accuracy with 30 training images. We used a simple nearest neighbor classifier at the top of the hierarchy. Experiments on the Caltech-101 dataset were performed primarily to make sure that we are within the range of reported accuracies. We share many of the concerns expressed by Pinto et al [61] that the Caltech-101 data set and the associated train/test protocols are not sufficiently informative of the overall recognition capability of a system. For this reason, we did not spend time optimizing the performance of our networks for this data set.

Caltech-101 images have low intra-category variation. Most of the images are centered and approximately of the same size. To see whether our system can handle large intra-category variations in gray-scale images, including translations and scale variations, we trained a network with 4 categories of images. These categories had a large amount of intra-category variation. The top of the network was exposed to over 10000 different training images. Figure S4 shows some examples of training images and Figure S5 shows some examples of test images for this network. On a hold out set, this network gave 92% accuracy. We also found that the network performs impressively in qualitative testing. A stand-alone demonstration of this network that lets users test their own images under different transformations is available for download from Numenta's website (http://www.numenta.com/about-numenta/demoapps.php). We are also happy to note that researchers outside Numenta have had success training recognition systems using HTMs. A case study on recognizing architecture drawings, including detailed parameter files for NuPIC software, is available at http://www.numenta.com/links/vision_exp.php

We have done a small set of experiments exploring the use of temporal information during inference. These experiments were performed on the Pictures data set. During inference, the network was shown a sequence of images. The first level of the network used the sequential information to compute the likelihood of Markov chains according to the equations we described in the Model section. We measured the recognition accuracy, on a frame-by-frame basis, while playing short (4 time frames) of translating inputs in a noisy background. The temporal boundaries where the input switched from one category to another were not marked or transmitted to the network. The recognition accuracy of the network that used temporal inference was up to 30% higher compared to the recognition accuracy obtained by a sliding window averaging (window length = 4) of frame-by-frame instantaneous recognition. More details on this experiment is available on Numenta's website (http://www.numenta.com/links/tbi_overview.php). This experiment is also available as part of the NuPIC software from Numenta. We have not done any studies incorporating temporal inference for grayscale image recognition or incorporating it at multiple levels of the hierarchy. These topics are currently under investigation and development.

**Figure 11:**
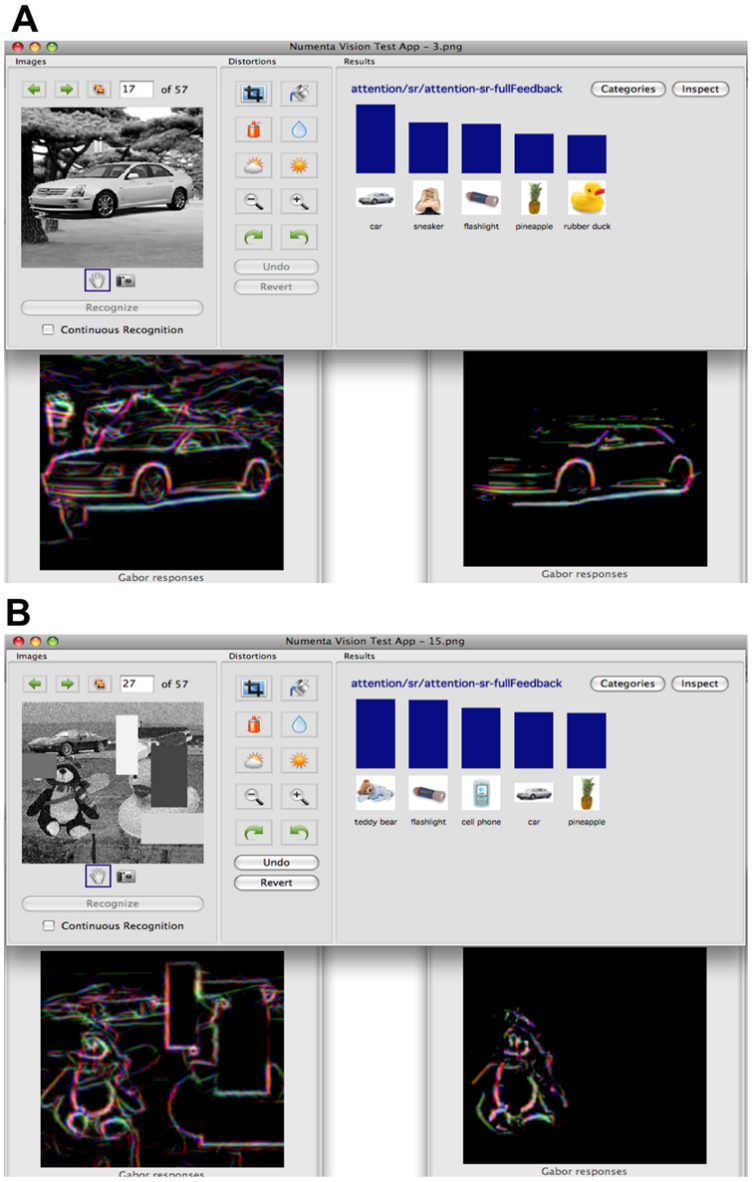
Top-down segmentation. Figures A and B show the effect of top-down propagation in HTM networks. The top half of each figure shows the original image submitted to the HTM, along with blue bars illustrating the recognition scores on the top five of the eight categories on which the network was trained. The bottom-left panel in each figure shows the input image after Gabor filtering. The bottom-right panel in each figure shows the image obtained after the feedback propagation of the winning category at the top of the HTM network. In these Gabor-space images, the colors illustrate different orientations, but the details of the color map are not pertinent. A). The input image has a car superposed on background clutter. The network recognizes the car. Top-down propagation segments out the car's contours from that of the background. B). The input image contains multiple objects superposed on a cluttered background and with some foreground occlusions. The network recognition result identifies teddy bear as the top category. Feedback propagation of this winning category correctly isolates the contours corresponding to the teddy bear.

We have also done experiments using feedback propagation in HTMs. The goal of these experiments was to verify that top-down propagation in HTMs can be used to locate and segment out objects in cluttered scenes with multiple objects. Figure 11 shows the results of inference and top-down propagation in a network that was trained on eight categories of images. During training, the objects were shown in isolation on a clean background. The test images contained multiple novel objects superposed on busy backgrounds. In most cases, one of the objects in the test image was the top result in the inference. Feedback propagation is initiated from the top of the network after the first flow of feed-forward propagation. After bottom-up propagation, the belief vector at the top of the network is modified such that the winning coincidence has strength one and all other coincidences have strength zero. This message is then propagated down in the network by combining with bottom-up information in the rest of the levels of the hierarchy. The resultant image obtained at the lowest level of the network isolates the contours of the recognized image from the background clutter and from other objects in the scene. These experiments show how top-down propagation in the current model can be used for segmentation, for the assignment of border-ownership, and for the ‘binding’ of features corresponding to a top-level hypothesis [62]. More examples of top-down propagation are available at http://www.numenta.com/links/top_down.php

### Example application: a model for the subjective contour effect

The cortical circuit described in this paper can be used for studying and modeling physiological phenomena. In this section, we report some preliminary positive results that we obtained modeling the *subjective contour* effect in visual inference [63] using these circuits. The primary goal of this section is to serve as a proof of concept for the possible applications of the circuit model. A detailed investigation of the subjective contours effect is beyond the scope of this paper.

**Figure 12:**
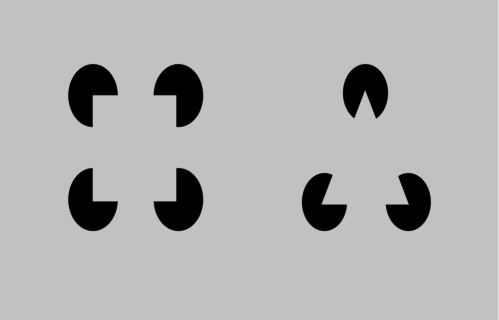
Kanizsa diagrams. A Kanizsa square (left) and a Kanizsa triangle (right) are shown.

The subjective contour effect is a well known cognitive and physiological phenomenon. Figure 12 shows examples of Kanizsa diagrams that produce this effect. When viewing such diagrams, humans perceive edges even in regions where there is no direct visual evidence for edges. Lee and Nguyen [64] found that neurons in area V1 responded to such illusory contours even though their feed-forward receptive fields do not have any evidence supporting the presence of a line. In addition to finding the neurons in V1 that respond to the illusory contours, Lee and Nguyen also studied the temporal dynamics of their responses. The summary of their findings is that the population averaged response to illusory contours emerged 100 milliseconds after stimulus onset in the superficial layers of V1 and at approximately 120 to 190 millisecond in the deep layers. The responses to illusory contours in area V2 occurred earlier, at 70 milliseconds in the superficial layers and at 95 milliseconds in the deep layers. These findings suggest that top-down feedback is used in the generation of illusory contours.

In [1], Lee and Mumford suggested this could be the result of Bayesian computations. Their argument was that the presented stimulus, according to the statistics of the visual world, is adequate to create a high-level hypothesis of the rectangle even though the edges are missing. The activation of this global hypothesis, at areas V2 and above, in turn constrains the activity of lower level neurons through the feedback messages. The HTM theory provides a mechanism for training a visual cortical hierarchy and the HTM circuit model gives a detailed anatomical circuit that can be used to test this hypothesis.

#### Subjective contour effect in HTMs

**Figure 13:**
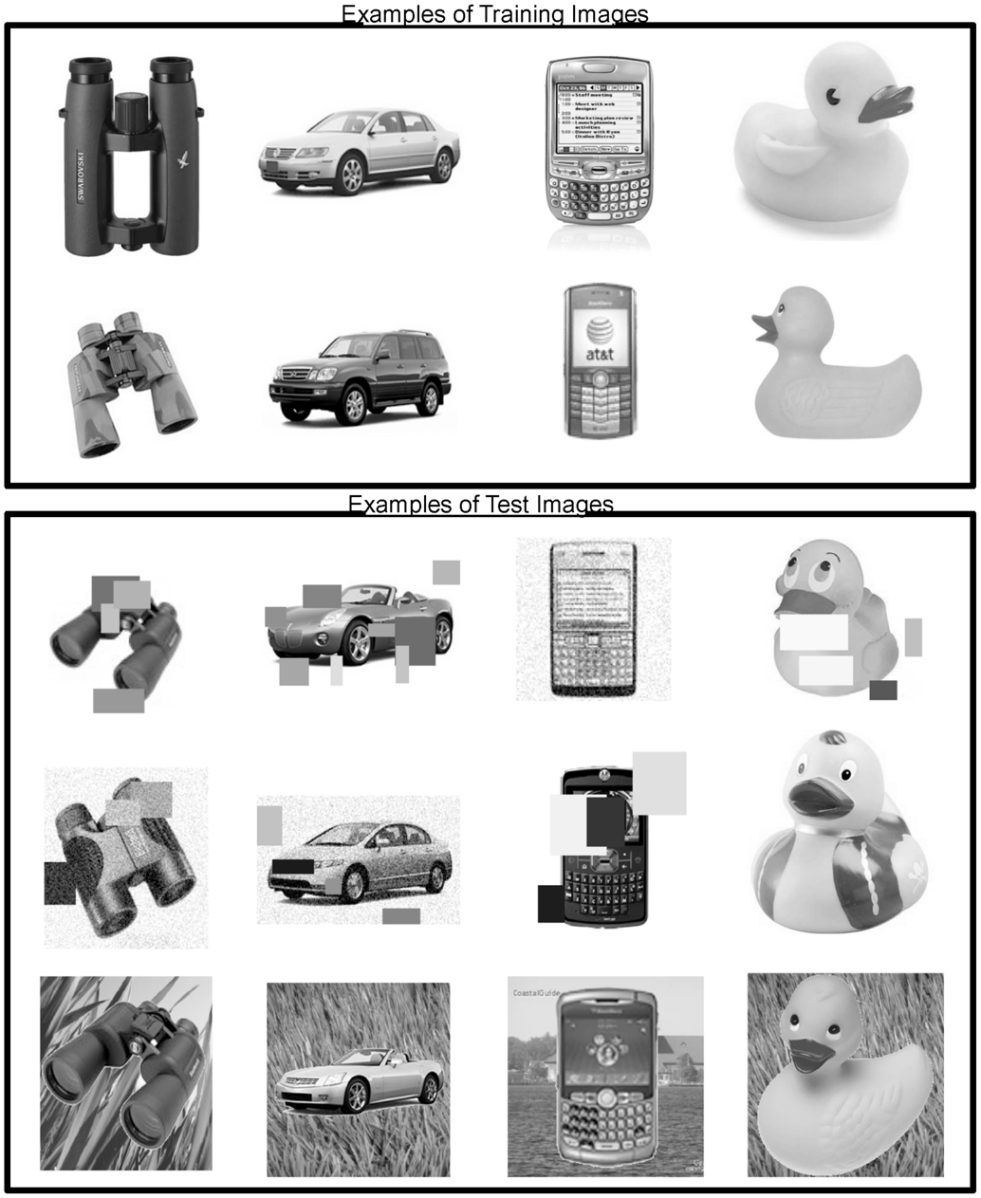
Examples of training and testing images for an HTM network trained for visual object recognition. The top two rows are examples of training images. The bottom three rows are examples of correctly recognized test images. The last row shows test images that incorporated distracter backgrounds.

We used Numenta's NuPIC software environment to train a visual pattern recognition HTM network on which we tested the subjective contour effect. We started with an HTM network that was trained to recognize four different categories of objects: binoculars, cars, cell phones, and rubber ducks. This network had a three level HTM hierarchy. Figure 13 shows examples of training and testing images for these categories. When presented with a test image, the output from the top-level node is a distribution that indicates the network certainty in different categories. In addition to recognizing input patterns, the HTM network can also propagate information down in the hierarchy using the belief propagation techniques that we described in earlier sections. Feeding information back in the hierarchy is used to segment the object from clutter and to locate the object in the image. More details about the training process for HTMs is available in [27] and in [8].

In this example the network recognizes a static visual image. This is a special case of the dynamic programming computations we described in that it uses only a single instant in time for inference. (An example that uses temporal inference to recognize time-varying patterns is available as part of the NuPIC software from Numenta. More details on this example are available at http://www.numenta.com/for-developers/education/tbi-overview.php.) HTMs need time-varying patterns to learn, and the general mode of operation is to perform inference on time-varying test patterns. However, in some problem domains such as image recognition, there is often sufficient information to perform inference without using time-changing patterns. In such cases, correct recognition can be obtained by a single feed forward pass through the network. This is consistent with observations about the speed of processing in the human visual system [65].

In order to perform the subjective contours experiment, we trained this network on an additional category: rectangles. This was done by presenting the network with a few images of rectangles during the training session. Only intact rectangle images were shown during training. The network then recognized novel rectangles of different aspect ratios.

**Figure 14:**
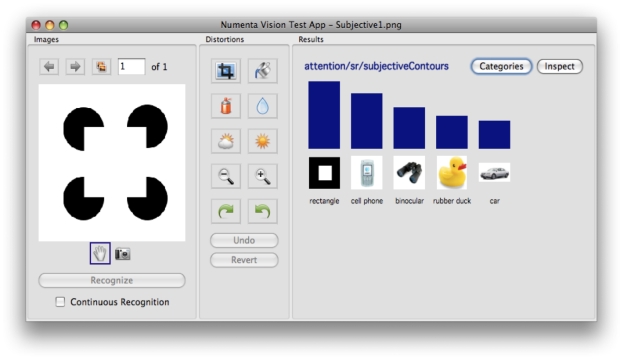
Recognition of the Kanizsa square by an HTM network. The network was not shown Kanizsa squares during training. The bar graph displays the order of recognition certainty of the HTM.

**Figure 15:**
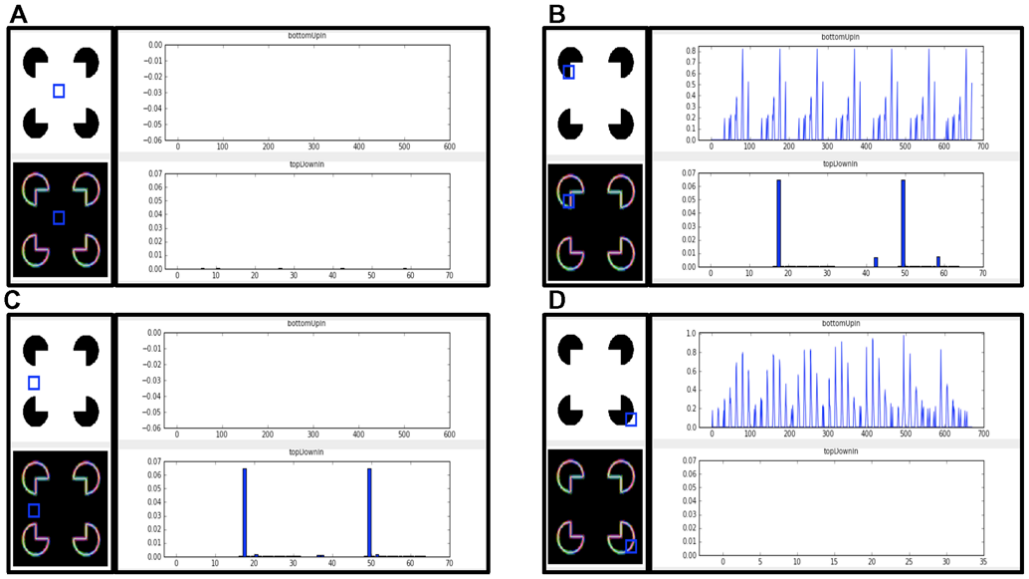
Subjective contour effect in HTM . Feed-forward and feedback inputs of 4 different nodes at level 1 of the HTM network for the Kanizsa rectangle stimulus. Four figures, (a) to (d), are shown corresponding to 4 different nodes from which the responses are recorded. In each figure, the left top panel is the input stimulus and the left bottom panel is the input stimulus as seen by the network after Gabor filtering. In these panels, the receptive field of the HTM node is indicated using a small blue square. In each figure, the top-right panel shows the feed-forward input to the node and the bottom-right panel shows the feedback input to the node. The feed-forward inputs correspond to the activity on thalamo-cortical projections. The feedback inputs correspond to the activations of the layer 6 cells that project backward from the higher level in the hierarchy. (a) The receptive field of this node does not contain any edges. There is no feed-forward input and no feedback input. (b) The receptive field of this node has a real contour in its input field. The node has both feed-forward and feedback inputs. (c) The subjective contour node. The receptive field of this node has no real contours. Therefore, the feed-forward input is zero. However, the feedback input is not zero because the network expects the edges of a rectangle. This is the subjective contour effect. (d) The opposite of the subjective contour effect. In this case, a real contour is present in the receptive field of this node but it does not contribute to the high-level perception of the rectangle. Hence the feedback input to this node is zero even though the feed-forward response is non-zero.

We then tested the network on a Kanizsa square test pattern. Figure 14 shows the response of the network to the Kanizsa square test pattern. The network classifies this pattern as a rectangle, even though this type of pattern was not seen during training. We examined the network for the presence of illusory contour responses. Illusory contour responses are characterized by top-down activations with no bottom-up activation. We used the capability of Numenta's software to inspect the node states of a network to probe for illusory contour responses. Figure 15 shows the feed-forward and feedback inputs to nodes at 4 different locations. The subjective contour effect can be seen in Figure 15(C). There are no actual contours in the receptive field of this node. Therefore, the feed-forward input of this node is zero. However, the feedback input is nonzero because the network expects the edges of a rectangle. This is the subjective contour effect.

**Figure 16:**
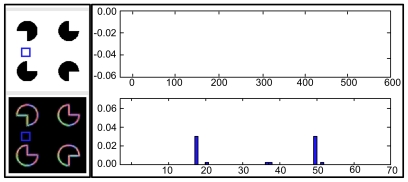
Reduced subjective contour effect. When presented with a corrupted version of a Kanizsa rectangle, the HTM still recognizes a rectangle but with reduced certainty. Shown are the feed-forward and feedback inputs to a node analogous to Figure 15(C). The node is receiving feedback indicating the network expects an edge at this location, but the strength of this expectation is substantially reduced compared to a non-corrupted rectangle.

We did an additional experiment where we presented a corrupted Kanizsa square identical to one of the control experiments used in [64]. As shown in Figure 16, the corrupted rectangle produces a subjective contour response similar to, but substantially weaker than, the one produced by an intact Kanizsa figure. This is consistent with the results that Nguyen and Lee saw in monkeys. In our experiment the corrupted figure was recognized as a rectangle at the top of the network, albeit with a lower level of certainty. This lower level of certainty is reflected in the lower activation level of the subjective contour. Had we put a threshold on the strength of recognition at the top level to filter out input images that were not close to any category, we could have reduced the subjective contour response to close to zero.

It is proposed that the delayed onset of the illusory contour response reported by Lee and Nguyen [64] occurs because of the delays caused by propagating messages up and down in the hierarchy. Lee and Nguyen also showed that the illusory contour response occurs first in the superficial layers and then in the deep layers. This is also consistent with the cortical circuit model in Figure 9 because the feedback information first reaches the Markov chain neurons in layer 2/3 (the yellow neurons in Figure 9) and then is integrated into the layer 5 neurons.

The subjective contour effect in our model was generated exclusively by feedback circuits. This is in contrast to models that rely only on lateral connections within a level for contour completion. For example, [66] uses a stochastic contour completion algorithm. However, using only local information completes contours that might not be in agreement with the higher-level perception. The use of top-down information is more in agreement with visual experience and with studies that suggest that visual understanding and awareness might be needed for the perception of illusory figures [67]. Lee and Mumford [1] suggested that the formation of illusory contours is primarily a top-down mechanism, as also suggested in our experiments, in combination with lateral mechanisms proposed in [66].

Using our cortical circuits theory, it is possible to study this phenomena in more detail. For example, it is possible to identify specific neurons in specific laminae and specific columns that will be active with top-down input and also to study their temporal characteristics. This is left as future work.

## Discussion

The mathematical model and biological mapping for cortical circuits derived in this paper is a starting point to achieve the final goal of a complete understanding of cortical circuits at least in prototypical sensory areas. We see three ways of advancing the circuits derived here: (1) Incorporation of more elements of HTM theory including learning, attention, actions, and asynchronous messages between levels, (2) Incorporation of more biological data including more detailed modeling of dendritic properties, and specific inhibitory cells, and (3) Incorporation of other constraints such as wiring length optimization and ease of learning. In the following subsections we discuss how a combination of the above factors could explain many aspects of cortical circuits that are not modeled in this paper.

### 

#### Learning mechanisms and inhibitory neurons

The circuits discussed in this paper have been mapped to the belief propagation equations in a learned HTM node. We have not discussed how the learning algorithms themselves can be analyzed for their biological plausibility. We saw in the Results section that some of the intra-columnar vertical connections required to support the belief propagation equations can be pre-wired because these connections do not depend on external stimuli. Most other connections, the ones representing sequence memories and coincidence patterns, are learned. Learning these connections requires mechanisms that support competition, inhibition and online learning.

Throughout this paper we have adopted the common assumption that excitatory neurons provide the prominent information-processing pathway and that inhibitory neurons largely play a supporting role in implementing the learning algorithms. This assumption is partially based on the fact that most of the inter-laminar and long distance connections within a cortical area are provided by the spiny excitatory neurons, whereas the smooth inhibitory interneurons more prevalently connect locally within their layer of origin. It is the excitatory cells that connect long distance in both vertical and lateral dimensions and their activity is then molded by local inhibitory neurons [15]. It is expected that inhibitory neurons will play a prominent role when biologically realistic mechanisms are considered for the learning of the HTM node states. Inhibitory mechanisms are required for competition during learning. Inhibitory neurons could also be required for avoiding instabilities produced by positive feedback loops.

#### Overlapping nodes and sparse representations

The HTM nodes described in this paper are shown as discrete entities with abrupt boundaries, which does not correspond to biology where overlapping receptive fields and imprecise boundaries are commonly found. The idealized HTM node instantiation gives us the flexibility to create mathematical abstractions that can be analyzed; however, it needs to be modified to make a full biological correspondence. One way to accommodate this could be to use HTM nodes with heavily overlapped input fields to construct a region. With overlapped input fields, the resultant network, viewed as a Bayesian network, has cycles in it. Although theoretical guarantees do not exist for the convergence of belief propagation in such hierarchies, successful systems have been built based on belief propagation in loopy graphs [32],[33] and our limited experience with implementing overlapping input fields have similarly shown no tendency for loop-induced failures.

The HTM model in this paper uses sparse-distributed representations [68],[69] when considering the representations within an entire hierarchical level. However, it does not use sparse-distributed representations within a node. In domains where a node is exposed to data that has rich characteristics, the model would require modifications to include sparse-distributed representations within an HTM node. This can be achieved by relaxing the assumption that a node represents a set of mutually exclusive hypotheses. We have made some recent progress with this formulation. However, this is beyond the scope of this paper.

#### Cortical maps

The circuits derived here attempt to explain only the information processing in a learned model. Any spatial arrangement of the columns of the circuit that preserves the connections between columns would still do the same information processing. Hence, the circuits here provide no explanation for observed cortical maps [70],[71].

We can think of several plausible reasons for the existence of cortical maps that are consistent with the circuits in this paper. One reason is that organizing the columns in a particular manner in space could reduce wiring length or some other resource that needs to be conserved. Another reason could be that a topographical organization of “similar” patterns could reduce the search space for coincidence-detection and sequence-learning algorithms. Circuits for implementing self organizing map [72] algorithms need to be incorporated into the theory. This work is left for the future.

#### Asynchronous message passing

The belief propagation messages in this paper were derived under the simplifying assumption that child-node states change synchronously. This assumption made the derivations and the circuits easier to understand. Our preliminary investigations indicate that relaxing this assumption may require additional communication between hierarchical levels which may explain the role of some of the layer 6 cells.

#### Attention mechanisms

As mentioned earlier, the circuits derived in this paper do not incorporate a detailed mechanism for attention control. Hypothesis-driven attention is an important aspect of perception and plays an important role in belief propagation as well [29]. It is known that thalamus plays an important role in cortico-cortical communication, acting as a dynamic control of information passed from one cortical area to another [57],[73]. There are multiple connections to the thalamus. There are feedback connections that control the gating of feed-forward information and the feed-forward connections through the thalamus are viewed as an alternate pathway to the direct cortico-cortical projections. There are computational reasons why all these pathways should exist. In belief propagation, the messages required for attention control are different from those of standard feedback messages. The attention control messages instantiate variables at intermediate levels and therefore affect the results of feed-forward propagation, whereas the standard feedback messages in belief propagation do not interact with feed-forward messages.

Some forms of attention can also be considered as an internal motor action because the attention control mechanism activates parts of the network and blocks the other parts. In that sense, the attention control mechanism can also be thought of as analogous to the 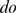 operator proposed by Pearl [74] to model the effect of actions in causal Bayesian networks. It is a tantalizing clue that Guillery and Sherman [75] found that the layer 5 pyramidal cells that project to the pulvinar of the thalamus also project to motor structures. Incorporation of the attention pathway into the derived circuits is left for future research.

#### Neuron biophysics and dendritic properties

Much is known about the properties and biophysics of dendrites, dendritic action potentials, and the biochemical pathways related to synapses [76]. The model presented here does not address most of this knowledge. We see the potential for extending the HTM model in these directions. Indeed, we believe the best way to understand the detailed properties of neurons is within the context of a larger scale theoretical framework.

#### Predictions of the theory

In this section we give a brief summary of potential predictions that can be generated from the theory.

- Different cells in layer 2/3 of the same cortical column (same bottom-up feature) will become active as part of different sequential contexts.
- Layer 2/3 contains two sets of pyramidal neurons. Neurons in one set deal exclusively with feed-forward processing and receive no feedback connections from higher levels and the neurons in the other set deal with the combination of feed-forward and feedback information. Unintended interactions between these two sets could produce cognitive defects.
- Long-range lateral connections in layer 2/3 encode sequential information. These connections can be altered by training with temporal patterns that have different statistics.
- Some vertical connections in a column pre-exist to provide a backbone for belief propagation computation. These can be wired using genetic information. We described several of these connections.
- A set of layer 5 cells represent the belief that combines both top-down and bottom-up influences for a stimulus. This prediction can be tested by examining the information represented locally in the layer 5 cells after the presentation of a stimulus that is locally ambiguous but globally coherent.
- Disabling feedback pathways will have the effect of confusing segmentation and border-ownership assignments. For example, subjective contour responses will get disrupted.
- With an appropriately defined training paradigm, top-down signals can predict missing/occluded bottom-up information, even when it cannot be predicted by local contour continuation.
- In the case of visual pattern recognition, smooth movement of the stimulus will increase recognition accuracy even in situations were background subtraction does not explain the effect. (For example, in a stimulus where background changes every instant in addition to the foreground object.)
- In cases where sequential information is required to disambiguate a stimulus, responses of layer 2/3 cells will become sparser (reduced activity) as more temporal information is accumulated.

#### Degrees of freedom and variations

Even with the combination of computational constraints and available anatomical data, several degrees of freedom remain in the mapping to biology. Because of this, the mapping to biology does not produce a unique circuit.

One source of variation can be found at the boundaries between lamina and between hierarchical regions. Let us consider the boundary between layer 4 and layer 3. The typical picture of layer 4 neurons is that they receive inputs from the thalamus and project to layer 3. However, a layer 4 neuron that projects to layer 3 can do the same computation even if that neuron is moved to layer 3 and if it receives direct bottom-up input from the thalamus. A similar degree of freedom exists between different levels of the hierarchy. For example, a neuron in layer 6 that sends feedback information to layer 2 of a child region can actually be moved to layer 2 of the child region.

These variations do not violate the computational principles we described and can be thought of as variations of the same theme. We believe that computational constraints will need to be combined with resource optimization constraints and physical constraints to completely understand why biology chooses some implementations over others. For example, some of these variations could be more advantageous than others for wiring length optimization [14]. It also is possible that these tradeoffs change with position in the hierarchy, the amount of convergence of bottom-up inputs, the need to send outputs and receive inputs from sub-cortical circuits, etc. The circuit derived in this paper provides a template to explore such variations.

### Conclusion

In this paper we have mapped a model of how the neocortex performs inference onto neocortical anatomy. The model, called Hierarchical Temporal Memory (HTM), is a type of Bayesian network which assumes a hierarchy of nodes where each node learns spatial coincidences and then learns a mixture of Markov models over the set of coincidences. The hierarchy of the model corresponds to the hierarchy of cortical regions. The nodes in the model correspond to small regions of cortex. We performed the mapping to biology in two stages. Starting with a mathematical expression of how each node performs inference, we created an abstract neuronal implementation. Next we mapped this abstract implementation onto observed anatomical data of cell types, cell layers, and micro-circuits in the cortex. We also showed results of an experiment where an HTM-based vision system exhibited the effects of illusory contours.

There are many unknowns and variations in cortical anatomy, and similarly there are many functions of the neocortex that are not accounted for by the HTM model. However, we believe the theoretical and anatomical constraints are sufficiently strong that the merger of the two is non-trivial and instructive. The ultimate goal of our work is to have a theoretical model of neocortex sufficiently tied to biological data so that the biology can lead to refinements of the theory, and the theory can lead to testable predictions about the biology. The work we have done, including that in this paper, suggests HTM is a good starting point for such a biologically grounded neocortical model.

## Supporting Information

Text S1 Derivation of belief propagation in HTM networks
(0.29 MB PDF)

Text S2 A toy example for belief propagation in HTM networks
(0.24 MB PDF)

Figure S1 The Pictures data set. The Pictures data set consists of 48 categories of binary line drawings. An example of each category is shown in the figure. Images are of size 32 pixels by 32 pixels. Training sequenes for HTMs are generated by animating these binary images with smooth translations and scale variations.
(0.05 MB PNG)

Figure S2 Learned Markov chain temporal groups. Figure shows a subset of the Markov chain temporal groups learned at the first level of the Pictures HTM network. The rows correspond to different Markov chains. The states of the Markov chains are shown as two-dimensional representations of their corresponding coincidence patterns. The connectivity between the elements of the Markov chains are not shown. The states within a Markov chain are perceptually similar even though their corresponding coincidence patterns are not similar in the pixel space.
(0.04 MB PDF)

Figure S3 Test examples for the table lamp category. These test images were generated by programmatically modifying the training images through translations, aspect ratio changes, pixel deletions and insertion of noise pixels.
(0.09 MB PDF)

Figure S4 Examples of grayscale training images. Figure shows examples of the training images used for training a 4 category HTM network. Most training images had an uncluttered background. The images presented to the network were of size 200 pixels by 200 pixels. The training images have a large amount of intra category variation in shape. In addition, the network was trained to recognize translations and scale variations of these categories.
(1.98 MB PDF)

Figure S5 Test images. Examples of test images used for the 4 category gray scale network. The test images were novel examples with significant variations in size and location in addition to the presence of background clutter.
(1.09 MB PDF)
