# Supplementary material for: Towards a Mathematical Theory of Cortical Micro-circuits
Source: PLoS Comput Biol. 2009 Oct 9;5(10):e1000532. doi: 10.1371/journal.pcbi.1000532 (PMC2749218; doi:10.1371/journal.pcbi.1000532)
Supplement: Text S1 — Derivation of belief propagation in HTM networks (0.29 MB PDF) [file pcbi.1000532.s001.pdf]

Text S1: Belief Propagation in HTM Networks

In this document we discuss the derivation of Bayesian belief propagation for HTM networks. Bayesian belief propagation was pioneered by Judea Pearl as an inference mechanism for Bayesian networks [1]. Like Bayesian networks, HTMs can be thought of as encoding relationships between random variables. Belief propagation is an approximate inference method for HTM networks.

Notation

We closely follow the notation used by Pearl [1]. The equations are described from the viewpoint of a node in the HTM network (see figure 1). We use ^-e to denote bottom-up evidence and ^+e to denote top-down evidence, from the node's view point. The bottom-up evidence at time t is denoted using ^-e_t and the sequence of bottom-up evidence from time 0 to t is denoted using $^-e_0^t$. Similar temporal indexing is used for the c variable representing the coincidence patterns in the node. The random variable representing the set of coincidence patterns in the node is C and the random variable representing the set of Markov chains in the node is G. The Markov chain transition probabilities are denoted using $P(C_t|C_{t-1}, G)$. We use $c(t)$ to represent all the possible coincidence patterns that can be active at time t and $c_i(t)$ to represent the event of coincidence pattern c_i occurring at time t. The top-down input message to the node at time t is denoted using π_t and the bottom-up output message from the node is denoted using λ_t. The top-down output messages of the node at time t are indicated using $\pi_t^{child\ node\ index}$ where the child node index refers to the destination child node. Similarly, the bottom-up input messages to the node are indicated using $\lambda_t^{child\ node\ index}$ where the child node index refers to the source child node.

Dynamic programming equations

In this section, we use dynamic programming [2] methods to derive the equations for sequential inference under some simplifying assumptions. Figure 2 shows the timing of the messages, as seen by node k. We

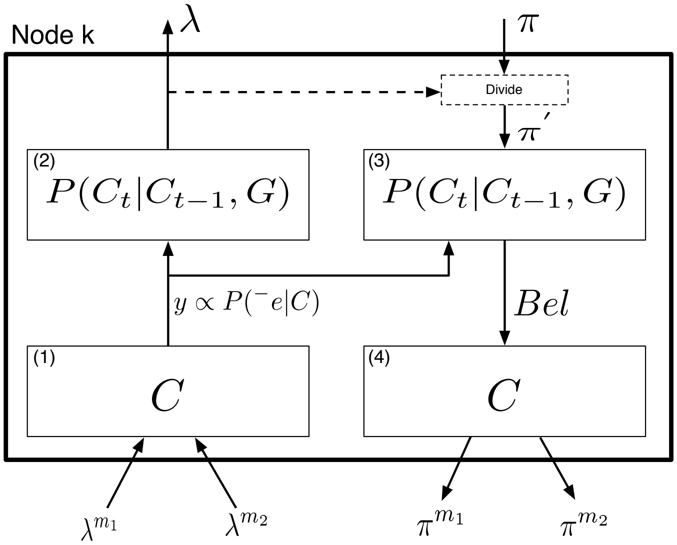

Figure 1. Block diagram of belief propagation computations in an HTM node.

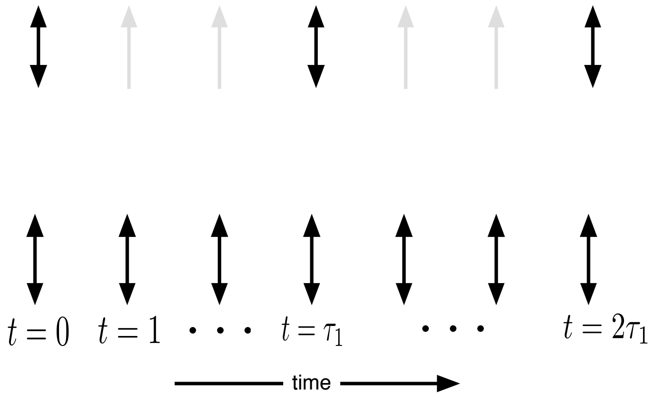

Figure 2. Timing of the input and output messages to Node k. The message passing between the node and its children occur at intervals of 1 time step, where as the message passing between the node and its parents occur at intervals of τ_k time steps.

assume that the bottom-up messages to the node arrive synchronized to time-steps $t = 0, t = 1$ etc. The messages from the parent node arrives at intervals that are multiples of τ_k, where τ_k is the time constant of this node. Similarly, it is assumed that bottom-up messages are passed at an interval of τ_k, although they are calculated for every time step $t = 0, t = 1, \cdots$.

Assume that a top-down message arrived at time $t = 0$, synchronous with bottom-up messages from the children. We derive the update equations for calculating the internal states and outputs of the node from $t = 0$ to $t = \tau_k$, as more bottom-up messages arrive. First, we derive the update equations for the belief state of the node.

$$\begin{aligned} Bel_t(c_i) &= P(c_i(t)|^- e_0^t, ^+ e_0) \\ &= (1/P(^-e_0^t|^+e_0^t)) \sum_{g_r \in G^k} \sum_{c_0^{t-1}} P(^-e_0^t|c_0^t, g_r, ^+ e_0) P(c_0^t, g_r|^+e_0) \\ &\propto \sum_{g_r \in G^k} \beta_t(c_i, g_r) \end{aligned} \quad (1)$$

where the dynamic programming variable β_t is defined as

$$\beta_t(c_i, g_r) = \sum_{c_0^{t-1}} P(^- e_0^t|c_0^t, g_r, ^+ e_0) P(c_0^t, g_r|^+e_0) \quad (2)$$

Then, the update equation becomes

$$\beta_t(c_i, g_r) = P(^-e_t|c_i(t)) \sum_{c_j(t-1) \in C^k} P(c_i(t)|c_j(t-1), g_r) \beta_{t-1}(c_j, g_r) \quad (3)$$

In the above equation, $P(^-e_t|c_i(t))$ denotes the likelihood of coincidence patterns based on evidence from below. This is calculated by multiplying the bottom-up output messages from child nodes according to:

$$P(^-e_t|c_i(t)) \propto \prod_{j=1}^{M} \lambda_t^{m_j}(r_i^{m_j}) \quad (4)$$

where coincidence-pattern c_i is the co-occurrence of $r_i^{m_1}$'th Markov chain from child 1, $r_i^{m_2}$'th Markov chain from child 2, $\cdots$, and $r_i^{m_M}$'th Markov chain from child M. This computation reflects the assumption that given the coincidence pattern the evidence from its components can be combined independently.

For the β update (equation 3) , the initial state is

$$\begin{aligned}\beta_0(c_i, g_r) &= P(^-e_0|c_i(t=0))P(c_i(t=0), g_r(t=0)|^+e_0) \\ &= P(^-e_0|c_i(t=0))P(c_i|g_r)P(g_r|^+e_0) \\ &= P(^-e_0|c_i(t=0))P(c_i|g_r)\pi_0(g_r) \qquad (5)\end{aligned}$$

The initial state incorporates $\pi_0(g_r) = P(g_r|^+e_0)$ – the message received from the parent at time $t = 0$. In the above equation, $P(c_i|g_r)$ is a learned conditional probability table that indicates the membership of each coincidence in the Markov chains of the node.

The top-down output messages that are sent to the child nodes indicate the node's degree of certainty in child nodes' Markov chains. (In non-loopy belief propagation, these messages are divided by the bottom-up messages from the children to avoid double counting.) This is done by converting the belief in coincidence patterns to the degree of certainty in Markov chains of child nodes based on the components of each coincidence pattern. The message to child m_i is calculated as

$$\pi^{m_i}(g_r) \propto \sum_i I(c_i)Bel(c_i) \qquad (6)$$

where

$$I(c_i) = \begin{cases} 1, \text{ if } g_r^{m_i} \text{ is a component of } c_i \\ 0, \text{ otherwise} \end{cases} \qquad (7)$$

and $g_r^{m_i}$ is the rth Markov chain in child node m_i.

The message for bottom-up transmission, $\lambda_t(g_r) = P(^-e_0^t|g_r(t))$, is calculated as follows:

$$\begin{aligned}
P(^-e_0^t|g_r(t)) &= \sum_{c_0^t} P(^-e_0^t, c_0^t|g_r) \\
&= \sum_{c_0^t} P(^-e_0^t|c_0^t)P(c_0^t|g_r) \\
&= \sum_{c_0^t} P(^-e_0^{t-1}|c_0^{t-1})P(^-e_t|c_t)P(c_0^{t-1}, c_t|g_r) \\
&= \sum_{c_0^t} P(^-e_t|c_t)P(c_t|c_{t-1}, g_r)P(^-e_0^{t-1}|c_0^{t-1})P(c_0^{t-1}|g_r) \\
&= \sum_{c_i \in C^k} P(^-e_t|c_i(t)) \sum_{c_j \in C^k} P(c_i(t)|c_j(t-1), g_r) \sum_{c_0^{t-2}} P(^-e_0^{t-1}|c_0^{t-1})P(c_0^{t-1}|g_r) \\
&= \sum_{c_i \in C^k} P(^-e_t|c_i(t)) \sum_{c_j \in C^k} P(c_i(t)|c_j(t-1), g_r)\alpha_{t-1}(c_i, g_r)
\end{aligned} \tag{8}$$

Where α is the dynamic programming variable whose update equation is given by

$$\alpha_t(c_i, g_r) = P(^-e_t|c_i(t)) \sum_{c_j(t-1) \in C^k} P(c_i(t)|c_j(t-1), g_r)\alpha_{t-1}(c_j, g_r) \tag{9}$$

The bottom-up output message is calculated as

$$\lambda_t(g_r) = P(^-e_0^t|g_r(t)) \propto \sum_{c_i(t) \in C^k} \alpha_t(c_i, g_r) \tag{10}$$

References

1. Pearl J (1988) Probabilistic Reasoning in Intelligent Systems: Networks of Plausible Inference. San Francisco, California: Morgan Kaufmann.

2. Howard RA (1960) Dynamic Programming and Markov Processes. Cambridge, Massachusetts: MIT Press.
