# Supplementary material for: Towards a Mathematical Theory of Cortical Micro-circuits
Source: PLoS Comput Biol. 2009 Oct 9;5(10):e1000532. doi: 10.1371/journal.pcbi.1000532 (PMC2749218; doi:10.1371/journal.pcbi.1000532)
Supplement: Text S2 — A toy example for belief propagation in HTM networks (0.24 MB PDF) [file pcbi.1000532.s002.pdf]

Text S2: A toy example for belief propagation in HTM networks

The figure below shows an example illustrating the qualitative meaning of belief propagation messages used in HTM networks. What is shown is a segment of a larger network that is used to recognize an input image. More details about the full network is available in [1, 2]. The software example and parameter files for constructing and training an HTM network on a line drawing data set is available as part of the NuPIC software that can be downloaded from http://www.numenta.com.

The shown network segment has two nodes at level 1 ($N^{1,1}$ and $N^{1,2}$) and one node ($N^{2,1}$) at level 2. Each node at level 1 has six coincidence patterns and two Markov chains. The coincidence patterns and Markov chain states of level-1 nodes are illustrated qualitatively to correspond to the visual patterns that occur in their receptive fields. The Markov chain $g1$ corresponds to a corner moving left and Markov chain $g2$ corresponds to a horizontal line moving upward.

Bottom-up messages are represented using shades of blue and top-down messages are represented using shades of green. Consider the distribution $y^{1,1}$ in node $N^{1,1}$. This represents the bottom-up likelihood of coincidence patterns in the node for the presented evidence. (For nodes directly attached to sensors this can be calculated, for instance, by treating the coincidence patterns as Gaussian centers.) Note that the distribution is peaked at $c4$ reflecting the presence of the corner in the node's receptive field. The output $\lambda^{1,1}$ is the bottom-up likelihood of the Markov chains in the node. This is shown to be peaked at $g1$. In general, this likelihood reflects the temporal history of bottom-up messages received by the node. However, since the corner coincidence pattern participates only in Markov chain $g1$, even an instantaneous Markov chain likelihood computation based on only the current coincidence pattern likelihood would still assign a higher value to $g1$ for this particular input pattern. On the other hand, the bottom-up Markov chain likelihoods of node $N^{1,2}$ are equal for the instantaneous coincidence likelihood, reflecting the fact that the horizontal line coincidence pattern participates in both Markov chains.

The higher-level node $N_{2,1}$ has stored two coincidence patterns. We assume that only these two coin-

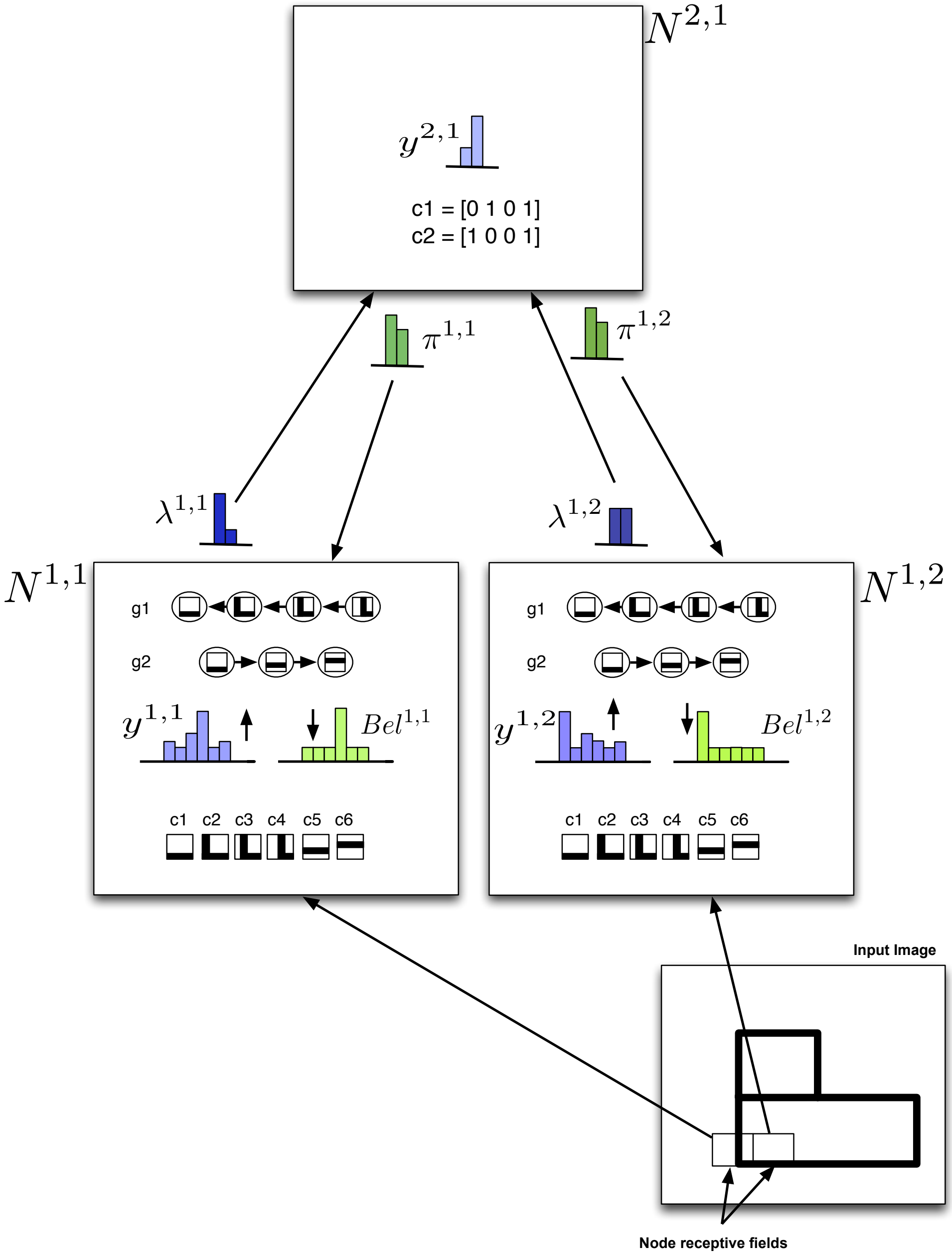

Figure 1. A toy example for belief propagation in HTM networks

cidence patterns occur in this toy world. Even though the message from child node $N_{1,2}$ was ambiguous about the Markov chain likelihoods, the coincidence pattern likelihood at $N_{2,1}$ is peaked for coincidence 2. This is because, among all the known coincidence patterns, coincidence pattern 2 is more likely given even the ambiguous inputs.

The peaked nature of the likelihood of coincidence patterns is reflected in the top-down message to child $N^{1,2}$. Even though child $N^{1,2}$ has ambiguous bottom-up information about its Markov chains, integrating more global information gives rise to a peaked top-down distribution in its Markov chains.

This is a qualitative example. The distributions shown in the diagram do not correspond to the result of any computation. The actual distributions computed will depend on the input strengths as well as on the relative frequencies of the coincidences and Markov chains.

References

1. George D, Jaros B (2007) The HTM Learning Algorithms. Numenta, Inc, Menlo Park, CA .

2. George D (2008) How the Brain Might Work: A Hierarchical and Temporal Model for Learning and Recognition. Ph.D. thesis, Stanford University.
