# Supplementary figures and images for: Towards a Mathematical Theory of Cortical Micro-circuits
Source: PLoS Comput Biol. 2009 Oct 9;5(10):e1000532. doi: 10.1371/journal.pcbi.1000532 (PMC2749218; doi:10.1371/journal.pcbi.1000532)

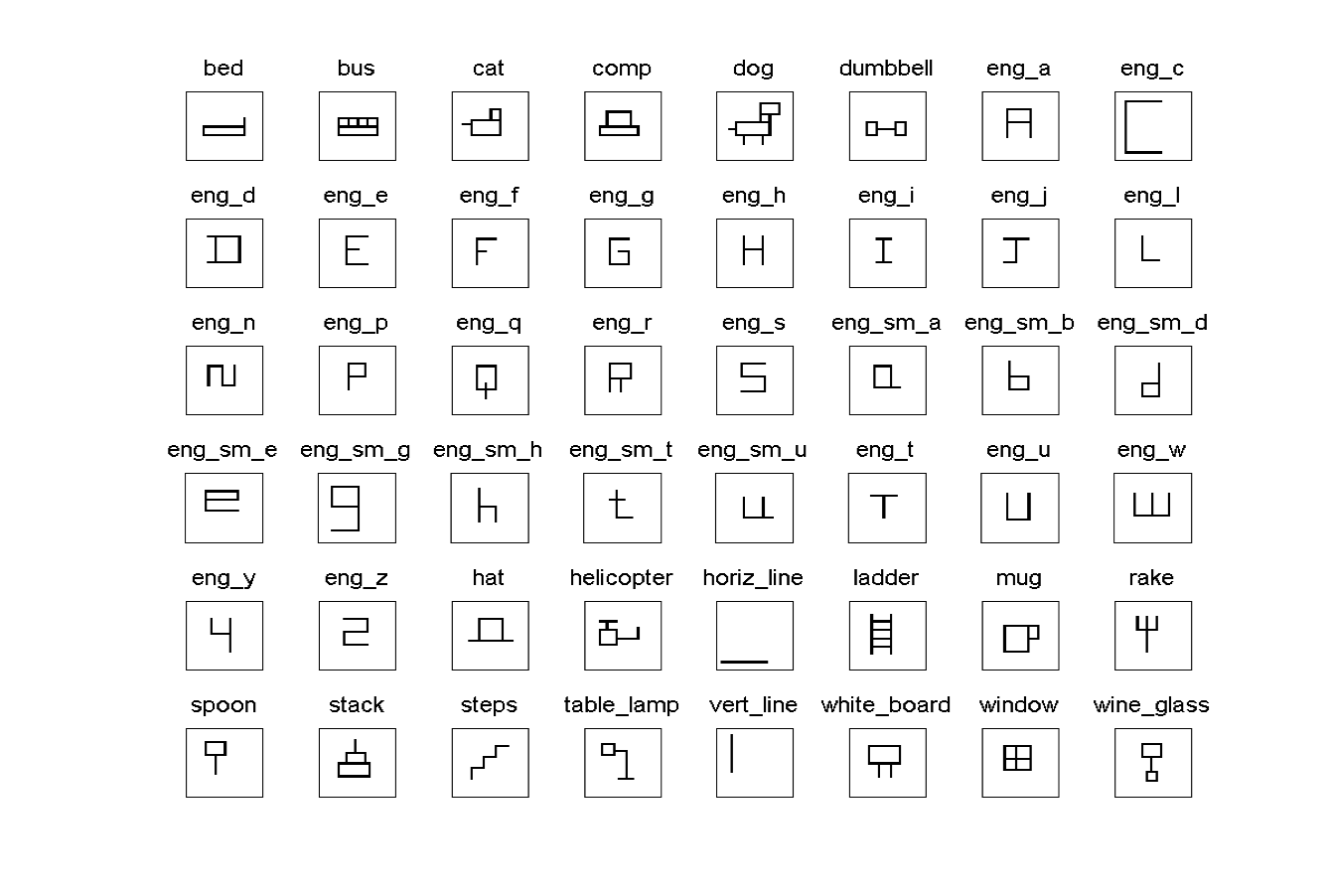

Supplement: Figure S1 — The Pictures data set. The Pictures data set consists of 48 categories of binary line drawings. An example of each category is shown in the figure. Images are of size 32 pixels by 32 pixels. Training sequenes for HTMs are generated by animating these binary images with smooth translations and scale variations. (0.05 MB PNG) [file pcbi.1000532.s003.png]

1

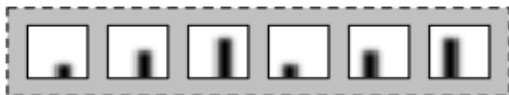

2

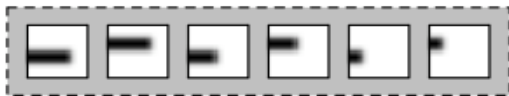

3

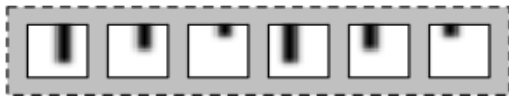

4

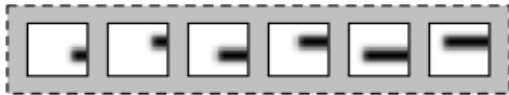

5

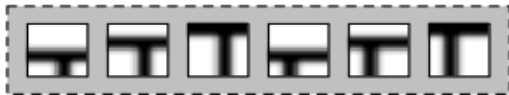

6

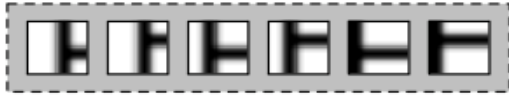

7

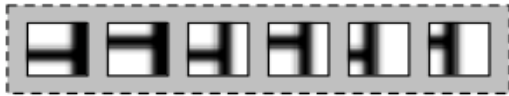

8

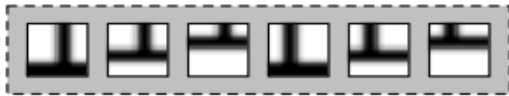

9

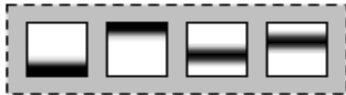

10

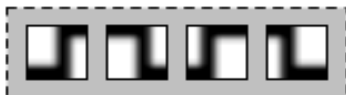

11

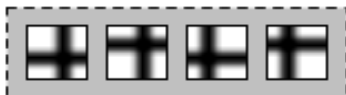

12

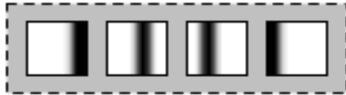

Supplement: Figure S2 — Learned Markov chain temporal groups. Figure shows a subset of the Markov chain temporal groups learned at the first level of the Pictures HTM network. The rows correspond to different Markov chains. The states of the Markov chains are shown as two-dimensional representations of their corresponding coincidence patterns. The connectivity between the elements of the Markov chains are not shown. The states within a Markov chain are perceptually similar even though their corresponding coincidence patterns are not similar in the pixel space. (0.04 MB PDF) [file pcbi.1000532.s004.pdf]

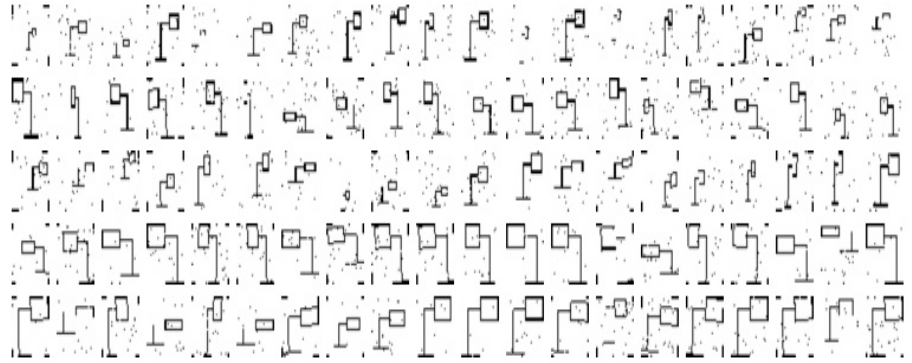

Supplement: Figure S3 — Test examples for the table lamp category. These test images were generated by programmatically modifying the training images through translations, aspect ratio changes, pixel deletions and insertion of noise pixels. (0.09 MB PDF) [file pcbi.1000532.s005.pdf]

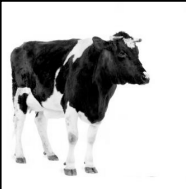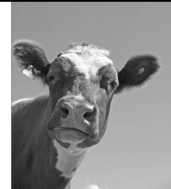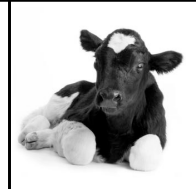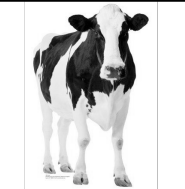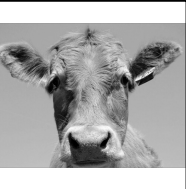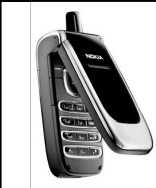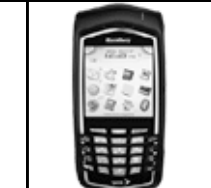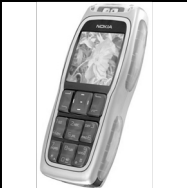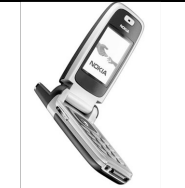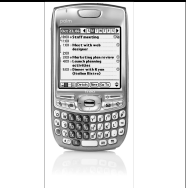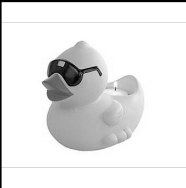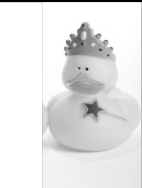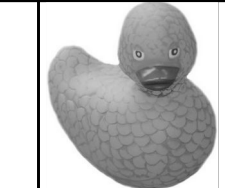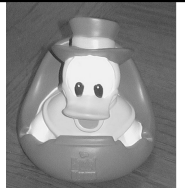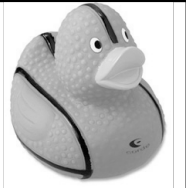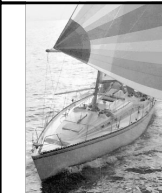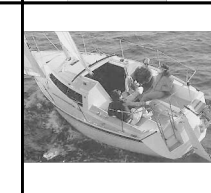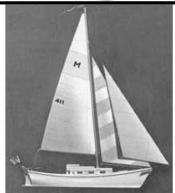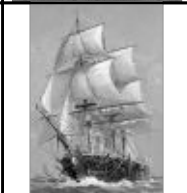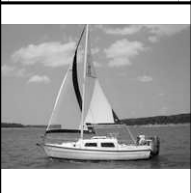

Supplement: Figure S4 — Examples of grayscale training images. Figure shows examples of the training images used for training a 4 category HTM network. Most training images had an uncluttered background. The images presented to the network were of size 200 pixels by 200 pixels. The training images have a large amount of intra category variation in shape. In addition, the network was trained to recognize translations and scale variations of these categories. (1.98 MB PDF) [file pcbi.1000532.s006.pdf]

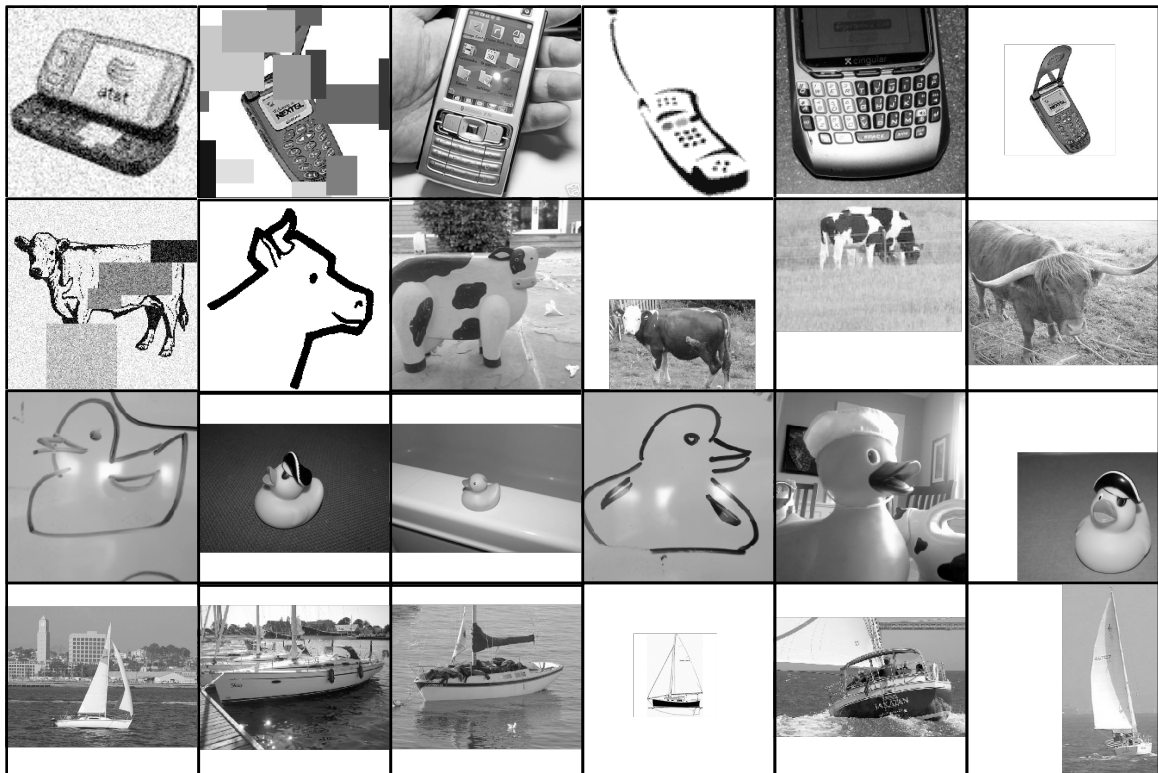

Supplement: Figure S5 — Test images. Examples of test images used for the 4 category gray scale network. The test images were novel examples with significant variations in size and location in addition to the presence of background clutter. (1.09 MB PDF) [file pcbi.1000532.s007.pdf]
